# Dependence of Bacterial Chemotaxis on Gradient Shape and Adaptation Rate

**DOI:** 10.1371/journal.pcbi.1000242

**Published:** 2008-12-19

**Authors:** Nikita Vladimirov, Linda Løvdok, Dirk Lebiedz, Victor Sourjik

**Affiliations:** 1 Interdisziplinäres Zentrum für Wissenschaftliches Rechnen (IWR), University of Heidelberg, Heidelberg, Germany; 2 Zentrum für Molekulare Biologie (ZMBH), University of Heidelberg, Heidelberg, Germany; 3 Zentrum für Biosystemanalyse (ZBSA), University of Freiburg, Freiburg, Germany; University of Illinois at Urbana-Champaign, United States of America

## Abstract

Simulation of cellular behavior on multiple scales requires models that are sufficiently detailed to capture central intracellular processes but at the same time enable the simulation of entire cell populations in a computationally cheap way. In this paper we present RapidCell, a hybrid model of chemotactic *Escherichia coli* that combines the Monod-Wyman-Changeux signal processing by mixed chemoreceptor clusters, the adaptation dynamics described by ordinary differential equations, and a detailed model of cell tumbling. Our model dramatically reduces computational costs and allows the highly efficient simulation of *E. coli* chemotaxis. We use the model to investigate chemotaxis in different gradients, and suggest a new, constant-activity type of gradient to systematically study chemotactic behavior of virtual bacteria. Using the unique properties of this gradient, we show that optimal chemotaxis is observed in a narrow range of CheA kinase activity, where concentration of the response regulator CheY-P falls into the operating range of flagellar motors. Our simulations also confirm that the CheB phosphorylation feedback improves chemotactic efficiency by shifting the average CheY-P concentration to fit the motor operating range. Our results suggest that in liquid media the variability in adaptation times among cells may be evolutionary favorable to ensure coexistence of subpopulations that will be optimally tactic in different gradients. However, in a porous medium (agar) such variability appears to be less important, because agar structure poses mainly negative selection against subpopulations with low levels of adaptation enzymes. RapidCell is available from the authors upon request.

## Author Summary

Chemotaxis plays an important role in bacterial lifestyle, providing bacteria with the ability to actively search for an optimal growth environment. The chemotaxis system is likely to be highly optimized, because on the evolutionary time scale even a modest enhancement of its efficiency can give cells a large competitive advantage. In this study, we use up-to-date experimental and modeling information to construct a new computational model of chemotactic *E. coli* and implement it in a computationally efficient way to simulate large bacterial populations. Our simulations are performed in a new type of attractant gradient that ensures a constant level of chemotactic excitation at any position. We show that optimal chemotactic movement in a gradient results from a fine balance between excitation and adaptation. As a consequence, steeper gradients require higher optimal rates of adaptation. Simulations demonstrate that the observed intercellular variability of adaptation times, which is caused by gene expression noise, may play a positive role for the bacterial population as a whole, by allowing its subpopulations to be optimally tactic in gradients of different strengths. We further show that optimal chemotactic properties in a porous medium (agar) are different from those in a liquid.

## Introduction

One of the central questions of modern systems biology is the influence of microscopic parameters of a single cell on the behavior of a cell population, a common problem in multi-scale modeling. In terms of bacterial chemotaxis, this issue can be formulated as the influence of signaling network parameters on the spatiotemporal dynamics of a population in various gradients of chemoattractants. The problem of efficient multi-scale simulation imposes strict requirements on the model: it should be maximally detailed to grasp the main features of the signaling network yet computationally cheap to simulate large numbers of bacteria.

Chemotaxis plays an important role in microbial population dynamics. Chemotactic bacteria in a nonmixed environment—that is in presence of nutrient gradients—have significant growth advantages, as shown experimentally for different bacterial species [1]–[4]. Modeling of microbial population dynamics indicates that motility and chemotactic ability can be as important for evolutionary competition as cell growth rate [5],[6].

*Escherichia coli* is an ideal organism for chemotaxis modeling, because of the rich experimental information collected over years of extensive research. In common with many other bacteria, *E. coli* can migrate towards high concentrations of attractants and away from repellents. In the adapted state, cells perform a random walk, which becomes biased in the presence of a spatial gradient. This swimming bias is based on temporal comparisons of attractant concentrations during cell runs. If the direction of a run is favorable, i.e. up the attractant gradient or down the repellent gradient, the run become longer. Between runs, the cell tumbles and reorients for the next run [7].

Chemotaxis in *E. coli* is mediated by an atypical two-component signal transduction pathway (for recent reviews see [8],[9]). Ligand molecules bind to clusters of transmembrane receptors, which are in complex with the histidine kinase CheA and the adaptor CheW. Each receptor can be either active or inactive, depending on ligand binding and the methylation level. The active receptor activates CheA, eliciting downstream phosphorylation of the response regulator CheY. Phosphorylated CheY (CheY-P) is dephosphorylated by CheZ. Receptors can be methylated by the methyltransferase CheR and demethylated by the methylesterase CheB, and methylation regulates the receptor activity. The methylation of receptors provides a sort of chemical ‘memory’, which allows the cell to compare the current ligand concentration with the past. Phosphorylation of CheB by CheA provides a negative feedback to the system, although it appears nonessential for exact adaptation [10],[11]. Phosphorylated molecules of CheY-P freely diffuse through the cytoplasm and bind to the flagellar motor protein FliM, causing motors to switch from CCW to CW rotation. Switching of the motors to the CW state results in a tumble and reorientation, whereas the CCW rotation corresponds to straight runs.

A number of mathematical models of chemotaxis have been proposed [10], [12]–[18], including two recent programs that simulate cell motion along with the intracellular pathway dynamics: AgentCell [19], which is based on the StochSim pathway simulator [20]–[22], and *E. solo* [23], which is based on the BCT simulator [24]–[26]. The current version of AgentCell (2.0) simulates the whole pathway stochastically, making it thus computationally very expensive. The *E. solo* program simulates the pathway using about 90 ordinary differential equations (ODEs). However, simulation of large bacterial populations on long time scales requires computationally cheaper models.

It was recently shown using fluorescence resonance energy transfer (FRET) that the amplitude of the initial CheY-P response can be described by a Hill function of a relative change in receptor occupancy during stepwise ligand stimulation [27]. Recent modeling efforts [12],[28],[29] show that a mixed-cluster Monod-Wyman-Changeux (MWC) model of strongly coupled receptors is consistent with the FRET data, and can account for the observed sensitivity and precise adaptation over a wide range of ligand concentrations. The amplitude of pathway excitation can therefore be determined using several algebraic equations describing the free energy of the cluster.

**Figure 1:**
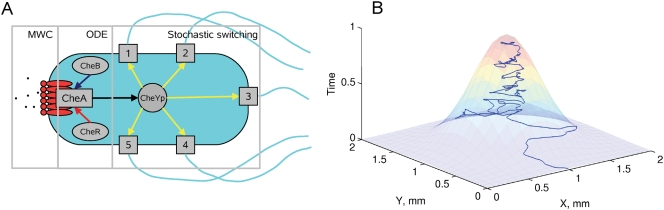
Model of chemotactic *E. coli*. (A) Scheme of the hybrid model. The activity of the receptor cluster depends on the local ligand concentration and the methylation level according to the MWC model. Methylation (red arrow) and demethylation (blue arrow) are performed by CheR and CheB. The phosphate group is transferred from active CheA to the response regulator CheY (black arrow). The concentration of CheY-P modulates the motor bias of 5 independent motors (yellow arrows), and their collective behavior makes the cell run or tumble. Ligand binding, receptors cluster switching, CheY phosphorylation and motor switching are considered to be in rapid equilibrium and are described by algebraic equations, while the methylation and demethylation kinetics are relatively slow and simulated using an ODE. Motor switching is simulated stochastically. (B) The model reproduces the swimming of *E. coli* cells up gradients of attractants, taking into account the effect of rotational diffusion. A typical path of a swimming virtual cell is shown in 2D space, with the relative time course shown along the Z axis, demonstrating how the cell finds the maximum attractant concentration and stays in its vicinity. The attractant concentration profile is overlayed.

In our model (Figure 1A), we employed the MWC model for a mixed receptor cluster [12] with a mean-field approximation for adaptation kinetics [30]. Due to its hybrid approach, the model allowed us to reduce the computational costs dramatically, while keeping the main quantitative characteristics of the cell response (methylation level, relative CheY-P concentration, motor bias) consistent with experimental data. To couple the bias of individual motors to the probability of tumbling, we applied a voting model for several independent motors, based on detailed experimental investigation of tumbling mechanics [31].

These components were combined into a new simulator for *E. coli* chemotaxis—RapidCell, which uses a hybrid pathway simulation instead of a fully stochastic or ODE approach, and is therefore computationally cheap. This allows the simulation of populations of 10^4^–10^5^ cells on a time scale of hours using a desktop computer.

To study the dependence of chemotaxis on gradient strength in a systematic way, we propose a new—constant-activity—gradient which ensures a constant average CheY-P level and cellular drift velocity along the gradient, in contrast to commonly used Gaussian and linear gradients. We show that the MWC model gives an approximately constant response over a wide range of ligand concentrations. Though purely theoretical, such a gradient serves as a perfect *in silico* assay to study the chemotactic properties of cells.

The chemotaxis pathway is robust to changes in network parameters and intracellular protein concentrations [10],[15],[32]. This enables efficient chemotaxis with varying levels of intracellular components and under perturbations from extracellular environment. However, adaptation time is not robust [10],[11],[33],[34] and varies even among genetically identical cells in a population because of stochastic variations in gene expression and low copy numbers of the adaptation enzymes.

Our simulations predict that in liquid media for any given gradient steepness, there is an optimal adaptation rate that provides the highest cellular drift velocity. We suggest a simple mechanism for this phenomenon: the optimal rate of adaptation is observed in a narrow range of kinase activity, where the average CheY-P level fits the operating range of the flagellar motor. In this range, the relation between CheY-P and motor bias is approximately linear, and cells perform chemotaxis with the highest efficiency.

The situation is different for cells swimming in agar. Here, the optimal range of motor bias appears to be very narrow and just slightly higher than in the non-stimulated state. Due to the porous structure of agar, cells with a higher CCW motor bias stay trapped for a longer time, thus negating advantage in chemotactic efficiency. This leads to a strong selection against cells which adapt slowly and therefore tend to overreact to chemotactic stimulation. On the other hand, chemotaxis in agar poses only a weak selection against cells with a high adaptation rate.

Our simulations suggest that in liquid media the variability in protein levels among cells may be advantageous for bacterial populations on a long time scales. In a nonmixed environment with different food sources and gradient intensities, such variability can help the whole population to respond to different gradients more readily, due to positive selection of subpopulations with optimal levels of adaptation enzymes in a given gradient.

## Methods

### Model of *E. coli* Signaling Network

#### MWC model

We applied the recently proposed MWC model for a mixed receptor cluster [12],[28],[29], which accounts for the observed experimental dose-response curves of adapted cells measured by *in vivo* FRET [27]. An individual receptor homodimer of type *r* (*r* = *a* and *s* for Tar and Tsr, respectively) is described as a two-state receptor, being either ‘on’ or ‘off’. Receptors form clusters with all receptors in a cluster either ‘on’ or ‘off’ together. The clusters are composed of mixtures of Tar and Tsr receptors. At equilibrium, the probability that a cluster will be active is [12]:
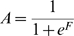
(1)where *F* = *F^on^*–*F^off^*, and *F^on/off^* is the free energy of the cluster to be on/off as a whole. For a cluster composed of *n_a_* Tar and *n_s_* Tsr receptors, the total free-energy difference is, in the mean-field approximation, *F* = *n_a_f_a_*(*m*)+*n_s_f_s_*(*m*), which is the sum of the individual free-energy differences between the two receptor states
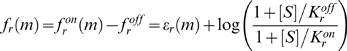
(2)where [*S*] is the ligand concentration, 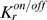 is the dissociation constant for the ligand in the on and off state, respectively. The methylation state of the receptor enters via the ‘offset energy’ *ε_r_*(*m*).

The model can also be generalized for binding multiple types of ligand [12],[28].

#### Adaptation model

Adaptation is modeled according to the mean-field approximation of the assistance-neighborhood (AN) model [12],[30]. Both CheR and CheB are assumed to bind receptors independent of their activity. A bound CheR (CheB) can (de-)methylate any inactive (active) receptor within the AN. Each bound CheR adds methyl groups at a rate *a*(1−*A*), and each bound CheB removes methyl groups at a rate *bA*. Under these assumptions, the kinetics in the AN model are given by
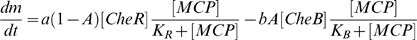
(3)We further assume that both enzymes work at saturation:
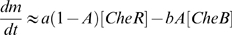
(4)Note that this equation does not imply a first-order reaction mechanism between the adaptation enzymes and receptors—the enzymes work in the zero-order regime. The linear products *a*(1−*A*)[*CheR*] (*bA*[*CheB*]) mean that a bound CheR (CheB) can only act if the receptor cluster is inactive (active), with probability (1−*A*) and *A*, respectively.

We further define the *relative adaptation rate k*:
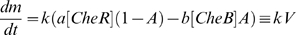
(5)The parameter *k* indicates the adaptation rate relative to the wild-type adaptation rate *V*. In the cells with normal steady-state activity (*A* ^*^ = 1/3), the rates and concentrations of the adaptation enzymes are related through *b*[*CheB*] = 2*a*[*CheR*]. In this work we assume that reaction rates *a* and *b* remain unchanged, and the variability in adaptation rate *k* is caused by variability in [CheR,CheB], provided that they change in a coordinated manner with the fixed ratio: [CheR]/[CheB] = 0.16/0.28 [35]. The latter ODE for methylation is integrated using the Euler method, so that the average methylation level evolves in time as
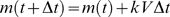
(6)To achieve high computational efficiency in the model, we assumed that the average methylation level *m* is a continuously changing variable within the interval [0,8], with linear interpolation between the key offset energies: *ε_r_*(0), 1.0; *ε_r_*(1), 0.5; *ε_r_*(2), 0.0; *ε_r_*(3), −0.3; *ε_r_*(4), −0.6; *ε_r_*(5), −0.85; *ε_r_*(6), −1.1; *ε_r_*(7), −2.0; *ε_r_*(8), −3.0, according to [12],[30].

#### Kinase activity

CheA kinase activity is assumed to be equal to the activity of the receptor complex (*A*). The differential equation for CheY-P is [32]

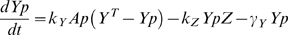
(7)Here *Yp* is [CheY-P], *Y^T^* — total [CheY], *Z^T^* — total [CheZ], *Ap* — active CheA, and *k_y_* = 100 *µM* ^−1^ *s* ^−1^, *k_Z_* = 30/[*CheZ*]*s* ^−1^, *γ_Y_* = 0.1 are the rate constants according to [32],[36],[37]. The rate of phosphotransfer from active CheA to CheY is much faster than the rate of CheA autophosphorylation (Table S1). Therefore, the concentration of CheY-P is obtained as a function of active CheA from the steady-state equation:
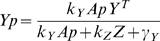
(8)In relative units, 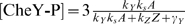, where *k_s_* = 0.45 is a scaling coefficient. The relative [CheY-P] = 0,1,3 correspond to fully inactive, adapted and fully active cluster, respectively. The absolute concentration relates to the relative as [CheY-P]*_abs_* = 3.1[CheY-P] (*µM*), following [38].

#### CheB phosphorylation

To study the effect of kinase-dependent CheB phosphorylation, we assumed that the concentration of phosphorylated (active) CheB follows the steady-state equation [15],[32]:
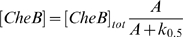
(9)where [*CheB*]*_tot_* is the total concentration of CheB (relative), and *A* is the kinase activity. In the steady state 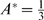 we assumed that 100%, 50%, or 25% of CheB can be phosphorylated, corresponding to [*CheB*]*_tot_* = 1,2,4 and 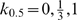, respectively. Note that at maximum kinase activity *A* = 1, the active [CheB] increases 1, 1.5 and 2 times compared to [CheR]; at steady state 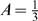 both enzymes have equal levels, whereas at positive chemotactic signal 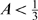 [CheB] is equal to [CheR] (*k*_0.5_ = 0) or lower than [CheR] (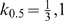).

#### Time-scale separation

We assume that the rates of ligand binding *t_l_*, rates of receptor-cluster conformational changes *t_k_* and receptor covalent modification *t_m_* are well separated in scales: *t_l_*≪*t_k_*≪*t_m_*. On our scale (∼1 s) the reactions of CheA autophosphorylation, phosphotransfer from CheA to CheY and CheB can be described as a rapid equilibrium state through algebraic equations. The slowest reactions—methylation by CheR and demethylation by CheB—are described through an ODE to reproduce the time scales of seconds and minutes required for adaptation. Table S1 shows the comparative rates of the main reactions.

#### Model verification

A summary of the parameters used in the model is given in Table 1, and a summary of models and assumptions is shown in Table 2. Along the lines of the MWC model for a mixed receptor cluster [12], we model a cluster of 18 receptors, composed of 6 Tar and 12 Tsr receptors. The catalytic rates *a* and *b* were chosen to achieve the proper time scale of adaptation according to *in vivo* FRET dose-response curves.

**Table 1:** Parameters used in RapidCell.

Parameter	Value	Reference
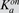	12 *µM*	Tar to Asp [21]
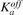	1.7 *µM*	Tar to Asp [21]
*K* ^*^(*K_D_*)	4.52 *µM*	Tar to Asp [14], this work
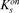	10^6^ *µM*	Tsr to (Me-)Asp [12],[29],[30]
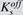	100 *µM*	Tsr to (Me-)Asp [12],[29],[30]
*n_a_*	6	[12]
*n_s_*	12	[12]
[*CheR*]	0.16 *µM*	wild-type level [35]
[*CheB*]	0.28 *µM*	wild-type level [35]
*a*	0.0625	this work
*b*	0.0714	this work
[*CheY*]*_tot_*	9.7 *µM*	[35]
*A* ^*^	1/3	[12],[30]
CCW *mb*_0_	0.65	[38],[41]
*H*	10.3	[38]
*v*	20 *µm* *s* ^−1^	[38]
*D_r_*	0.062 *rad* ^2^ *s* ^−1^	[7],[42]
Δ*t*	0.01 *s*	this work

**Table 2:** Models used in RapidCell.

Model	Reference
Receptor free energy: 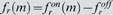 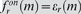 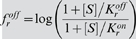	[12], [28]–[30]
Cluster free energy, in the mean-field approximation:*F* = *n_a_f_a_*(*m*)+*n_s_f_s_*(*m*)	[12],[29]
Cluster activity: 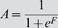	[12], [28]–[30]
Rate of receptor methylation, AN-model at saturation: 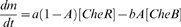	[12],[30]
Steady-state CheY-P concentration: 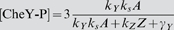	[32]
CCW motor bias: *mb* = (1+(1/*mb*_0_−1)(*CheYp*)*^H^*)^−1^	[38],[41]

As shown previously in [12],[29],[39], the MWC model for a mixed receptor cluster correctly reproduces the *in vivo* FRET response amplitudes to step-wise addition and removal of MeAsp [27],[40]. We also compare our model output with the published FRET response (Figure S1A), and show that the simulation is in good agreement with experiment, both for the amplitude and the duration of the chemotactic response. However, the steepness of the adaptation curve after attractant removal can only be roughly described by the existing model of CheB activity, a deficiency which needs to be addressed for more precise modeling in future.

The spatially extended StochSim model gives lower response amplitudes compared to FRET experiments [14]. Comparison of RapidCell and StochSim responses to addition and removal of Asp is shown in Figure S1B. The adaptation rate of StochSim seems very high compared to FRET experiments and RapidCell simulations (*k* = 8 times higher than the wild-type rate), which suggests that RapidCell will be much more sensitive to gradients than AgentCell [19].

RapidCell also reproduces experimental data on tethered cell stimulation with pulse and step changes of Asp concentration [41] (Figure S2A and S2B). The adaptation times after a step increase of *α*-methylaspartate (MeAsp) concentration over three orders of magnitude agree with experimental data reported in [33] (Figure S2C).

### Model of *E. coli* Motion

During a run, the cell is assumed to move with a constant speed *v* = 20 *µm*/*s*, while the direction of motion is affected by rotational diffusion [7],[42]. After each time step, the running direction is changed by adding a stochastic component with normal distribution 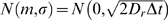 and diffusion coefficient *D_r_* = 0.062 *rad* ^2^ *s* ^−1^ [42].

#### Motor switching

The relative concentration of the response regulator [CheY-P] is converted into motor bias using a Hill function [38] (Table 2). Motor bias is the mean fraction of CCW rotation time for a motor: *mb* = *T_ccw_*/(*T_ccw_*+*T_cw_*), where *T_ccw_* and *T_cw_* are the means of exponentially distributed CCW and CW intervals, respectively. The equation
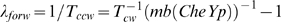
(10)gives the frequency of the Poisson process of CCW→CW motor switching. The frequency of reverse switching CW→CCW is *λ_rev_* = 1/*T_cw_*. After each time step Δ*t*, the motor can switch its direction from the present state, according to the current switching frequency *λ_forw_*_(*rev*)_, with probability *P_forw_*_(*rev*)_ = *λ_forw_*_(*rev*)_Δ*t*.

#### Runs and tumbles

Run and tumble events include the complex interplay of filaments in a bundle, the details of which have been investigated experimentally [31],[43]. To simulate the run and tumble behavior of a cell with several motors (*N* = 3–7) we consider the voting model, where the majority of the motors determines the cellular behavior.

***Model of voting motors.*** The cell has *N* = 5 motors switching independently, and the state of the cell is determined according to a voting model [13],[31],[44]. The cell switches from ‘Run’ to ‘Tumble’, if at least 3 of its 5 motors rotate CW, and from ‘Tumble’ to ‘Run’, if at least 3 of the 5 rotate CCW. The choice of *N* = 5 is arbitrary, and similar results are obtained for *N* = 3,7 under the condition of majority voting.

For model validation, simulations of cells with *N* = 3,5,7 motors were carried out (Table 3). The simulated run times (1.04–1.11 s) agree with the experimental value of 1.24±1.16 s [45]. The simulated tumble times (0.26–0.44 s) appear higher than the measured 0.14±0.08 s [7],[31]. However, the latter study [31] shows that the full tumble time, from bundle breaking in the old run to bundle consolidation in the new is 0.43±0.27 s. This estimate of tumble time reflects not only cell reorientation, but also the interplay of flagella and the resulting drop in cell speed, and the voting model reflects specifically this kind of tumble time estimate. The model with 5 motors is used in the following as default.

**Table 3:** Simulated run and tumble times for cells with different number of motors. Parameters: *T_ccw_* = 1.33 s, *T_cw_* = 0.72 s, *mb* = 0.65, *n* = 10000.

*N* Motors	Voting Threshold	*T_run_*	*T_tumble_*
3	2	1.11	0.44
5	3	1.09	0.33
7	4	1.04	0.26

***Tumbling angle.*** The tumbling angle is distributed according to the probability density function *f*(Θ) = 0.5(1+*cos*Θ)*sin*Θ, 0<Θ<*π* [46],[47], with *M*(Θ) = 67.5° which is close to the experimental measurement of 68° [7], and the corresponding shape of the function (Figure S3).

### Model of the Environment

The virtual cells are swimming in a 2D environment with a predefined attractant concentration field *S*(*x*, *y*, *t*). The domain geometry is either rectangular or circular, with reflecting walls. The simulation time was chosen to be short enough to avoid boundary effects. The rectangular domain is within (0, *x_max_*)×(0, *y_max_*), and the circular domain within (0, *r_max_*), with *x_max_* = *y_max_* = 2*r_max_* = 20 *mm*.

#### The constant-activity gradient

The gradients used in chemotaxis modeling are usually linear, Gaussian or exponential [19],[23]. However, in these gradients the signal is non-constant, which means it is strong at low attractant concentrations, and weak at high concentrations due to receptors saturation. Such a non-uniform distribution of the signal makes it difficult to estimate chemotactic efficiency over long time intervals—cells soon become ‘blind’ because receptors are saturated, and chemotactic drift decreases.

According to the MWC model, an increase in ligand concentration Δ*S* causes an initial rise in receptor free-energy difference
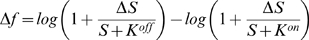
(11)Using the Taylor-series approximation,
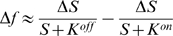
(12)leads us to the following approximation for free energy per concentration change:
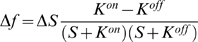
(13)


#### Simplified solution

The denominator in Eqn. 13 can be simplified by assuming
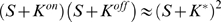
(14)and the unknown *K* ^*^ can be found from equation
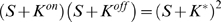
(15)

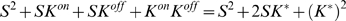
(16)

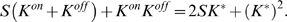
(17)which gives two alternative estimates for *K* ^*^: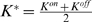 and 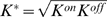, i.e. the arithmetic and geometric means of *K^on^* and *K^off^*.

At zero or relatively low ligand concentrations, the geometric mean has a high impact in Eqn. 17, and is preferable as an estimate. Indeed, in earlier work it was earlier referred to as the apparent dissociation constant *K_D_* of ligand binding [14]. However, at high concentrations, the arithmetic mean will have a higher impact in Eqn. 17, so it can be used as an alternative estimate. Our simulations indicate that within four orders of aspartate concentration the geometric mean serves as the best estimate of *K* ^*^ (Figure S4).

Taken together, the energy difference is approximated by 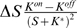. The differential equation
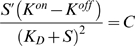
(18)describes the unknown function *S*(*x*), which will give the ‘constant-activity’ gradient shape. The function *S*(*x*) will give a constant change of energy difference *C* per length unit *dx* of cellular path along the gradient. In other words, such a shape of gradient will give a constant cluster activity at any ligand concentration.

Within the accuracy of a constant term, the latter differential equation was previously used by Block and Berg in [48], who derived it assuming that receptor occupancy is proportional to *S*/(*S*+*K_D_*), with a single *K_D_* for active and inactive receptors. The authors assumed that the chemotactic response is proportional to the change in receptor occupancy [27],[48]. They simplified this equation to reduce the variability of the 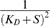 term, leading to the exponential form of the solution.

However, we can solve Eqn. 18 analytically:
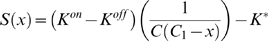
(19)where 
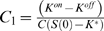
 is the constant of integration, determined by the initial condition *S*(0). The condition *S*(0) = 0 gives the following chemoattractant function:
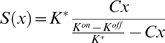
(20)


#### Constant-activity gradient of Asp

In the case of aspartate (*K^on^* = 12, *K^off^* = 1.7, *K* ^*^ = 4.52 *µM*), the *S*(*x*) function reads:
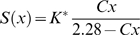
(21)Our simulations demonstrate that this form of constant-activity Asp gradient gives a constant cluster-activity response with reasonably good precision (see Results).

#### Gradient steepness

A cell swimming with speed *v* along the axis *X* from the point (*x* = 0) senses the monotonically increasing function *S*(*x*) and a constant change in receptor free energy
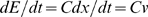
(22)per second, which is defined as the *steepness* of the constant-activity gradient.

#### Limiting condition

Note the necessary condition (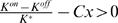) for Eqn. 20 to avoid singularity and negative concentrations. It sets the upper limit 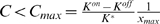 for the gradient steepness *C* within the domain (0, *x_max_*). For example, within a domain of size *x_max_* = 10 *mm*, the maximum steepness of a gradient of aspartate is *Cv* = 2.28/*x_max_v* = 4.56×10^−3^.

#### Constant-activity and exponential ramps

In contrast to spatial gradients, which direct the cellular motility in a certain direction, time ramps are used to study the chemotactic response of tethered cells [41],[48].

The constant-activity ramp of Asp was simulated according to Eqn. 20:
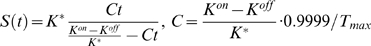
(23)with simulation time *T_max_* = 1000 seconds. The resulting value of *C* gives the maximum ligand concentration *S*(*T_max_*) = 9999*K* ^*^.

**Figure 2:**
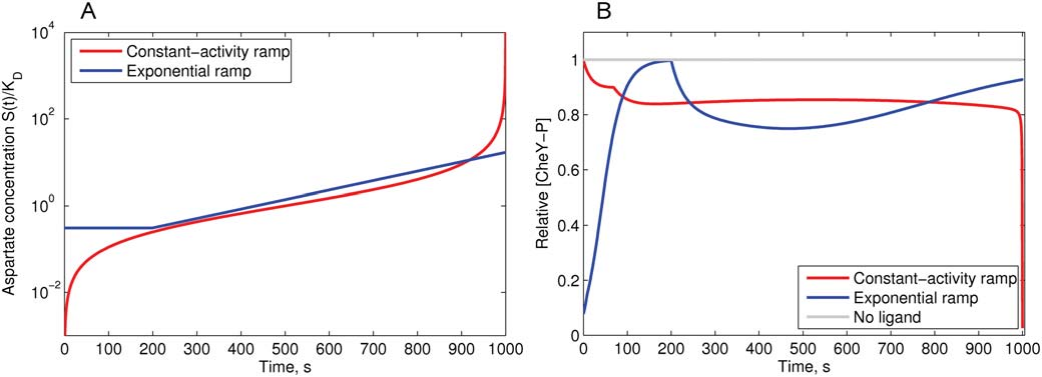
Simulation of the MWC model response to the constant-activity and exponential ramps of aspartate. (A) The concentration profiles of constant-activity and exponential ramps of aspartate, relative to *K_D_* = 4.52 *µM* (logarithmic scale). (B) The response of the MWC model to the applied constant-activity and exponential ramps. Upon stimulation with the constant-activity ramp, the [CheY-P] rapidly goes down during initial excitation—the single non-smooth point on the slope is the result of the piece-wise linearity of the methylation energy function. The constant-activity ramp produces a long flat response up to a concentration of 100*K_D_*, above which Tsr receptors become sensitive to the ligand and the cluster activity falls. Upon stimulation with the exponential ramp, the cell initially adapts to the minimum concentration *C_min_* = 0.31*K_D_*, and after 200 s the exponential ramp begins. After 700 s, adaptation overcomes excitation and [CheY-P] slowly returns to the steady state. Relative adaptation rate *k* = 1.

The exponential ramp was simulated as:
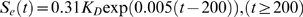
(24)after 200 s of adaptation to the initial stimulus 0.31*K_D_*, following the model and experiments of [48]. The concentration profiles are shown in Figure 2A.

#### Constant-activity gradient simulations

**Figure 3:**
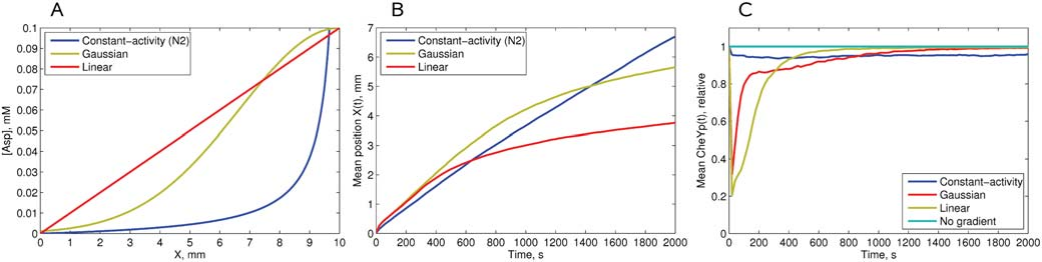
Simulations of chemotaxis in different gradients. (A) Concentration profiles of the gradients used in the simulations. (B) Chemotactic drift of cells in these gradients. The average position 〈*X*〉 of the cells is shown as a function of time. A population of 2000 cells starts moving from the left wall (*x*_0_ = 10 *µm*, *y*_0_ randomly distributed in (0, *y_max_*)), and swims for 2000 s. (C) Relative CheY-P concentration as a function of time, averaged over 2000 cells in the same gradients. The gray line indicates the fully adapted state [CheY-P] = 1.0 in a medium without attractant. Relative adaptation rate *k* = 1. All cellular parameters are as described in Table 1.

The constant-activity gradient (Eqn. 20) has an intensity 
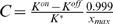
, and the domain has a rectangular (0, *x_max_*)×(0, *y_max_*) or circular (0, *r_max_*) shape. The gradient has its minimum *S* = 0 at *x* = 0 (or *r* = 0) and reaches its maximum *S* = 999*K* ^*^ at *x* = *x_max_* (or *r* = *r_max_*) (Figure 3A). In most simulations we used the circular gradient *S*(*r*), and the cells start swimming in random directions from the center *r* = 0.

#### Comparative set of constant-activity gradients (N1, N2, N3)

The circular constant-activity gradient (*r_max_* = 10 *mm*) has steepness *dE*/*dt* = *Cv* = 4.56×10^−3^. A set of other constant-activity gradients was obtained by changing the steepness by a factor of two: (1.14, 2.28, 4.56, 9.11, 18.22, 36.44, 72.88)×10^−3^. We further compare the chemotactic efficiency in three of them with moderate steepness (2.28, 4.56, 9.11)×10^−3^, and designate them as constant-activity gradients N1, N2 and N3. In other words, they are radially symmetric and have the form
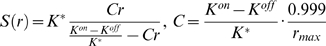
(25)with *r_max_* = 20,10,5 mm for N1, N2 and N3, respectively.

#### Linear gradient

We use a linear gradient *S*(*x*) = *Kx*, *x*∈(0,10 mm) with coefficient *K* = 10^−8^ *M* *µm* ^−1^ = 10^−2^ *mM* *mm* ^−1^ (Figure 3A).

#### Gaussian gradient

Another form of gradient we used is Gaussian *S*(*x*) = 10*K* exp(−(*x*−10)^2^/(2*σ* ^2^)), with shape parameter *σ* = 3.33 and the same coefficient *K* = 10^−2^ *mM* *mm* ^−1^ (Figure 3A).

#### Chemotactic efficiency

**Figure 4:**
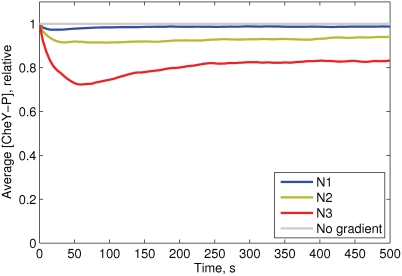
Average CheY-P levels of 5000 cells swimming in the constant-activity gradients N1 (blue), N2 (green) and N3 (red). Relative adaptation rate *k* = 1. The cell parameters are as described in Table 1.

Chemotactic efficiency was estimated as the average drift velocity of a cell population, measured between 200 and 500 s of simulation time, in the three basic constant-activity gradients N1, N2, N3. As shown in Figure 4, within this interval the average CheY-P level of cells is constant, and the drift velocity can be accurately measured by a linear fit.

#### Population behavior

The population behavior in the absence of attractant fits the diffusion equation 〈*r* ^2^〉 = 4*Dt*. Simulations give a diffusion coefficient *D* = 2.56×10^−6^ *cm* ^2^ *s* ^−1^, in agreement with the experimental *D* = 2.5–3.8×10^−6^ *cm* ^2^ *s* ^−1^ (see [45] and the review of other published values therein).

### Program RapidCell

The output file of the RapidCell program contains the key characteristics of the intracellular state (CheY-P level, methylation state, motor bias) and the geometric characteristics of cell motion (position and orientation). The model was implemented using Java classes similar to AgentCell [19], but with simplified architecture. The algorithm is implemented as a discrete-time Monte Carlo scheme with time step Δ*t* = 0.01 *s*. For random-number generation, we used external Java libraries [49],[50]. The code was written using Eclipse SDK (www.eclipse.org). The output data were analyzed with MATLAB (The MathWorks, MA).

#### Computational costs

Extensive computations of the chemotaxis signaling pathway are avoided in RapidCell due to the hybrid description of the signaling network. This leads to a dramatic drop in computational costs. For example, simulation of 1000 s long walk of a single cell in a ligand gradient takes only 1 s to run in RapidCell, compared to 133 minutes for AgentCell (based on StochSim without receptor coupling), while the spatially extended version of StochSim requires several days on the same hardware (Intel Pentium 4 CPU 2.40 GHz, RAM 1 GB, OS Linux Suse 10.2). Simulation of 1000 s long series of step responses with the BCT program—the core simulator of *E. solo*—takes 100 s under similar conditions (PowerPC G5, 1.8 GHz, RAM 1 GB, MacOS X).

RapidCell is platform-independent and runs as a console application. Its implementation provides a computational speedup of 8000 times compared to AgentCell (based on StochSim without receptor coupling), and approximately 100 times compared to BCT. It enables simulations of up to 100,000 cells to be completed within a time frame of hours using a desktop computer with comparable CPU power and RAM to those mentioned above.

### Experimental Methods

#### Strains and plasmids

*E.coli* strain RP2867 (*tap cheR cheB*) is a derivative of RP437 [51]. Plasmid pVS571 encodes *cheR* and *cheB-eyfp* as parts of one operon under control of a *pBAD* promoter and native ribosome binding sites. The insert *cheR cheB-eyfp* was recloned with *Sac*I and *Xba*I from the plasmid pVS145 which was constructed by cloning a PCR-amplified fragment containing *cheR* upstream of *cheB-eyfp* in the pVS138 plasmid [52] using a *Sac*I site introduced by the upstream PCR primer and a *Hin*dIII site in *cheB*.

#### Swarm experiments in soft agar plates

Tryptone-broth (TB; 1% tryptone, 0.5% NaCl) soft agar plates were prepared by supplementing TB with 0.27% agar (Applichem), 34 *µg* *ml* ^−1^ chloramphenicol, and indicated concentrations of arabinose. Cells were inoculated from fresh colonies grown on LB agar plates. Swarm assays were performed at 34°C for 10 hours or at 30°C for 17 hours. Following incubation, photographs of plates were taken using a Canon EOS 300 D camera, and subsequently analyzed with ImageJ (Wayne Rasband, NIH) to determine the diameter of the swarm rings.

#### Quantification of gene expression

For quantification of mean expression levels of the fluorescent reporter protein CheB-YFP, cells were grown in liquid TB medium supplemented with 34 *µg* *ml* ^−1^ chloramphenicol, and indicated concentrations of arabinose. Fluorescence was determined in a population of cells using flow cytometry on a FACScan (BD Biosciences) equipped with a 488 nm argon laser [32],[52]. The autofluorescence background was measured for control cells and subtracted from all values. Single-cell levels of fluorescent reporter proteins in swarm assays were measured by fluorescence imaging on a Zeiss AxioImager microscope and quantified with an automated custom-written ImageJ plugin [52].

To calibrate the fluorescence intensity in FACS and imaging data, a PerkinElmer LS55 luminescence spectrometer was used to determine the absolute number of reporter proteins in control cells. The cells were sonicated with a Branson Sonifier 450 until complete lysis was achieved and YFP fluorescence was measured at 510 nm excitation and 560 nm emission. Sonicated cells without a fluorescence reporter were used as a negative control, and their autofluorescence was subtracted from all values as background. A solution of purified YFP of known concentration, determined by Bradford assay and absorbance measurement by a Specord205 spectrophotometer (Analytik Jena), was used to produce a calibration curve, relating fluorescence to molecule number. Cell number in 1 ml culture was counted using a Neubauer counting chamber, and cell volume was determined by measuring cell width and length by imaging. These values from one culture were used to provide a conversion factor from FACS or imaging values to single-cell protein levels.

## Results

### Chemotaxis in Different Gradients

To test our model (Figure 1A), we compared cellular behavior in the proposed universal constant-activity gradient with other gradients, observing the single cell swimming (Figure 1B) and the behavior of large populations. The key characteristics we consider are the CheY-P concentration and the drift velocity along the gradient.

#### Response of the MWC model to ramps

It was previously shown that tethered cells respond with constant strength to an exponentially rising gradient of MeAsp, in the range between 0.31 and 3.2*K_D_* [48]. We simulated the response of the MWC model to increasing ramps of Asp in the exponential and constant-activity form (Figure 2A). Indeed, the exponential ramp gives nearly constant response between 0.5 and 3.0*K* ^*^, consistent with the model of [48].

However, the constant-activity ramp results in a chemotactic response that remains approximately constant over three orders of ligand concentration—between 0.1 and 100*K_D_* (Figure 2B). If Tsr is non-sensitive to the ligand, constant activity remains up to 1000*K_D_*. However, since Tsr receptors are able to respond to aspartate non-specifically, the activity drops to zero, as previously shown for a mixed-receptor cluster [12],[27].

#### Chemotactic efficiency of cell populations in different gradients

To study chemotactic efficiency in common gradients that arise from general diffusion models, we simulated chemotactic motility in linear and Gaussian gradients (Figure 3A), and compared them with the constant-activity gradient. The chemotactic efficiency was estimated by the average drift velocities of populations consisting of 1000 identical cells. In Figure 3B, one can see that in the linear and Gaussian gradients the drift velocity decays after about 400 and 800 s, respectively, indicating that cells loose sensitivity due to receptor saturation. In contrast, the constant-activity gradient keeps the drift velocity constant at any point (Figure 3B), as expected.

This population behavior can be explained by the intracellular CheY-P levels of the cells in these gradients. Gaussian and linear gradients result in a strong excitation at low attractant concentrations, and poor excitation at high concentrations (Figure 3B). In contrast, the constant-activity gradient produces an approximately constant level of CheY phosphorylation across the cell population (Figure 3C). These two unique properties of the constant-activity gradient—constant drift velocity and constant average CheY-P—favor this gradient as a reliable *in silico* assay to study the chemotactic motility of cells.

#### Average CheY-P level in the constant-activity gradients

Simulation of cell populations in the constant-activity gradients N1, N2 and N3 demonstrate that the average CheY-P level depends on gradient steepness and remains stable over long time intervals (Figure 4). These three gradients were used further, as default, to measure chemotactic efficiency under different test conditions.

### Optimal Adaptation Rates in a Liquid Medium

**Figure 5:**
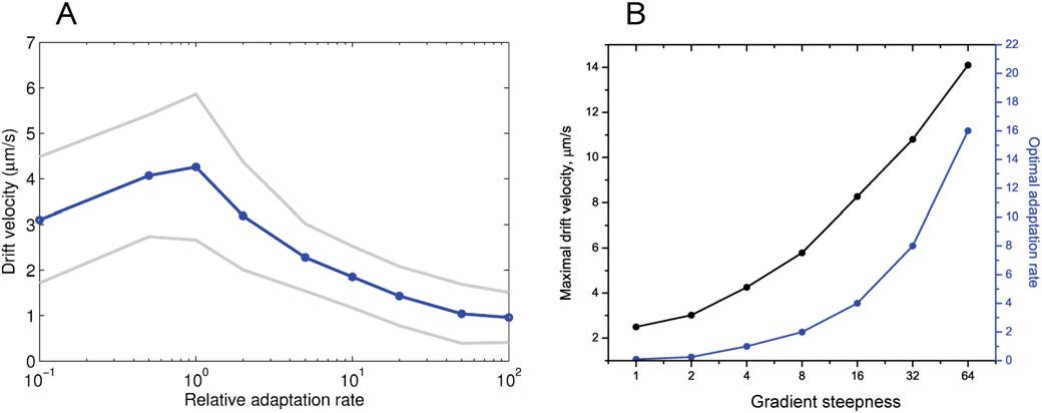
Chemotactic properties of cells at different adaptation rates in constant-activity gradients. (A) Drift velocity of cells in the constant-activity gradient N2 as a function of adaptation rate. The horizontal axis shows the adaptation rate *k* relative to the wild type (logarithmic scale). Gray lines show standard deviations. (B) Maximal drift velocities (black) and the corresponding optimal adaptation rates (blue) as a function of gradient steepness. The steepness of the constant-activity gradients was changed over a 64-fold range, as described in the section ‘Model of the environment’.

We used the constant-activity gradient to study the effect of adaptation rate on chemotactic efficiency. For this purpose, we simulated homogeneous populations consisting of cells with the same adaptation rate. In a fixed constant-activity gradient, the population drift velocity depends on adaptation rate in a unimodal manner (Figure 5A). A zero level of adaptation enzymes (non-adapting cells) results in a low drift velocity, though it is clearly distinguishable from non-chemotactic behavior. A proportional increase of adaptation rate improves cellular drift velocity up to a certain maximum, after which it slowly declines again. Extremely high adaptation rates, more than 100 times higher than wild-type, make the cells non-chemotactic (Figure 5A).

To study chemotactic efficiency as a function of gradient steepness, cells were simulated in six constant-activity gradients with the steepness changing 64-fold, from 1.14 to 72.88×10^−3^, (Figure 5B). In each gradient, we determined the optimal adaptation rate, at which cellular drift velocity reaches its maximum. The simulated drift velocities are in the same range as those measured experimentally for *E. coli* in steep gradients (7 *µm* *s* ^−1^) [53]. Our simulations indicate that experimental cell-drift velocities are inlikely to exceed 15 *µm* *s* ^−1^, corresponding to an extremely steep and short-scale gradient. In very weak gradients, the drift velocity can be as low as 2.5 *µm* *s* ^−1^, still distinguishable from the non-chemotactic cellular drift (0.8 *µm* *s* ^−1^). Interestingly, we observed that the optimal adaptation rate rises in proportion with the gradient steepness (Figure 5B).

**Figure 6:**
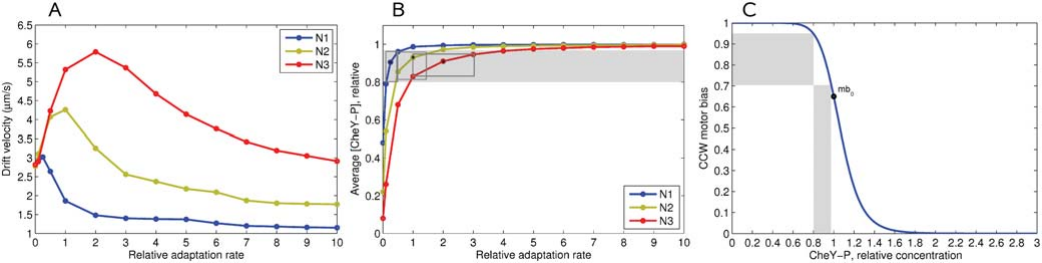
Optimal chemotactic behavior at different adaptation rates. (A) Drift velocities of cells as a function of adaptation rate, in the constant-activity gradients N1 (blue), N2 (green), N3 (red). For each adaptation rate, the drift velocity was estimated from the simulation of 1000 cells, with standard error of mean 0.05. (B) Average CheY-P levels of cells in the same simulations. Black dots indicate the adaptation rate at which drift velocity is maximal. Gray rectangles show the intervals of optimal adaptation rates, defined by taking the 90%-interval from the drift velocity maximum. The width of each rectangle indicates the optimal adaptation-rate interval, and height shows the corresponding CheY-P interval. All three intervals of adaptation rates fall into the same CheY-P interval: [0.80,0.97], shown by the gray band. (C) The CCW motor bias as a function of CheY-P. Gray bands indicate the optimal CheY-P interval and the corresponding operating range of the motor. The cell parameters are as described in Table 1.

To investigate the latter effect in more detail, we varied the adaptation rate from 0 to 10-fold relative to the wild-type. In steeper gradients, the optimal adaptation rate is indeed higher (Figure 6A), and the peak of the drift velocity becomes less sharp. To find the reason for the observed dependence between the gradient steepness and optimal adaptation rate, we tracked the average CheY phosphorylation levels of the virtual cells. As one can see in Figure 6A and 6B, in all gradients the 90%-intervals around the velocity peaks correspond to adaptation rate intervals [0.1,0.5], [0.4,1.5], [1,3], respectively. These three intervals fall into to the same interval [0.80≤CheY-P≤0.97], within the error of estimation. The optimal adaptation rates which give maximal drift velocities correspond to an average [CheY-P]∼0.9. In steep gradients, the profile of average CheY-P flattens, and the optimal adaptation rate becomes higher (Figure 6B).

The reason why the interval [0.80≤CheY-P≤0.97] corresponds to optimal chemotaxis is evident from the profile of motor bias as a function of CheY-P (Figure 6C). The interval [0.80≤CheY-P≤0.97] corresponds to the operating range of the motor [0.95≥*mb*≥0.72], where the dependence between *mb* and CheY-P is approximately linear. In this interval, chemotactic behavior is most efficient in liquid media. The optimal adaptation rate therefore sets the CheY-P level to fit the motor operating range. In steep gradients, the adaptation rate must be high enough to balance the strong excitation and set CheY-P within this optimal interval. In shallow gradients, adaptation must be slow enough to allow excitation, otherwise the cells become adapted before they are able to respond.

### Effect of [CheR] to [CheB] Ratio on Chemotactic Efficiency

**Figure 7:**
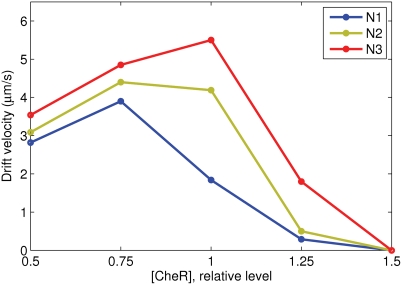
Effect of variable [CheR] on chemotactic efficiency. The vertical axis shows drift velocities. The level of [CheB] is fixed at the wild-type value (0.28 *µM*), while [CheR] is varied relative to wild type (0.16 *µM*). Note the steep fall in the drift velocities for [*CheR*]>1, where the steady-state CheY-P produces tumbling behavior.

The effect of varying the [CheR] to [CheB] ratio was studied at fixed [CheB] in three constant-activity gradients N1, N2, and N3 in a liquid medium. The chemotactic efficiency dramatically decreases above [*CheR*] = 1 (Figure 7), because the resulting higher steady-state CheY-P level produces tumbling behavior. Below [*CheR*] = 1, chemotactic efficiency decreases slowly for N3, or goes up for the N1 and N2 gradients. The latter effect is caused by a shift of average CheY-P level to the optimal interval, where the chemotactic sensitivity is the highest due to a more optimal fit to the motor operating range.

### Effect of CheB Phosphorylation on Chemotactic Efficiency

**Figure 8:**
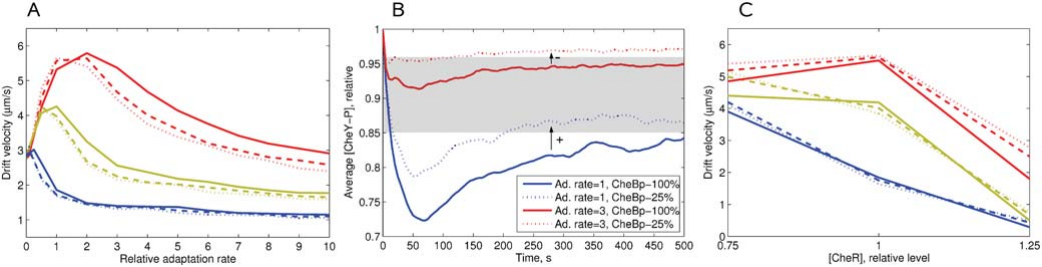
Effect of CheB phosphorylation on chemotactic efficiency in a liquid medium. (A) Drift velocity as a function of adaptation rate in the constant-activity gradients N1 (blue), N2 (green), N3 (red). The ratio of [CheR] to [CheB] at steady state is left as in the wild type (0.16/0.28), ensuring the steady-state activity *A* ^*^ = 1/3 in all cases. Solid lines correspond to cells with 100%-active CheB at steady state, dashed lines - 50%-active, finely dashed - 25%-active CheB. (B) The average [CheY-P] resulting from the balance between CheR and CheB activity determines the positive or negative role of CheB phosphorylation. Cells are simulated in the gradient N3, at adaptation rates of 1.0 and 3.0. Kinase-dependent CheB activity means that CheB works more weakly at *A*<1/3, and thus the average [CheY-P] is higher than the level obtained for constantly active CheB. Such a shift improves chemotaxis at low adaptation rates, but reduces it at high rates. The optimal range of CheY-P is shown by the gray band. (C) Drift velocities at variable [CheR] and variable CheB activity and fixed [CheB] (0.28 *µM*, wild type). Solid, dashed and finely dashed lines indicate 100%, 50% and 25% active CheB, respectively. Adaptation rate *k* = 1, other cell parameters as described in Table 1.

We have further studied the effect of CheB phosphorylation feedback on chemotactic efficiency in a liquid medium. Under the assumption that [CheR] and [CheB] perfectly match each other (*A* ^*^ = 1/3), the CheBp-effect is positive when the adaptation rate is lower than the optimum, and negative when the adaptation rate is higher, in the given gradient (Figure 8A). This effect is caused by the reduction of CheB activity relative to CheR, when the kinase activity *A* is below the steady-state level (*A* ^*^ = 1/3), as described in the section ‘Model of *E. coli* Signaling Network’. The average CheY-P level is thus shifted up, which results in a positive or negative effect of CheB phosphorylation, depending on the rate of adaptation (Figure 8B).

The positive role of phosphorylation can be significantly increased when the ratio of [CheR] to [CheB] is non-perfect (Figure 8C). For example, 25%-active CheB can significantly counteract the strong negative effect of [CheR] = 1.25 in the N3 gradient—the drift velocity rises from 1.8 to 2.8 *µm* *s* ^−1^ (55%). At [CheR] = 0.75 the effect is not so dramatic, but remains significant—the average drift velocities increase by about 10–15% in all three gradients. This suggests that CheB phosphorylation helps to maintain chemotaxis at fluctuating concentrations of CheR and CheB, when their ratio is not perfect due to gene-expression noise.

### Swarm Plate Simulations

In the swarm assay in soft agar, bacteria consume an attractant, thereby creating a local gradient, and follow it in the form of a growing ring [54],[55]. We assume that the intensity of the moving gradient remains constant, and use the constant-activity gradient as a simple model for the swarm assay simulation. The constant-activity gradient provides a constant cellular-drift velocity at any distance from the center of the plate. This property allows us to use it as a stationary model of the real moving gradient of attractant.

**Figure 9:**
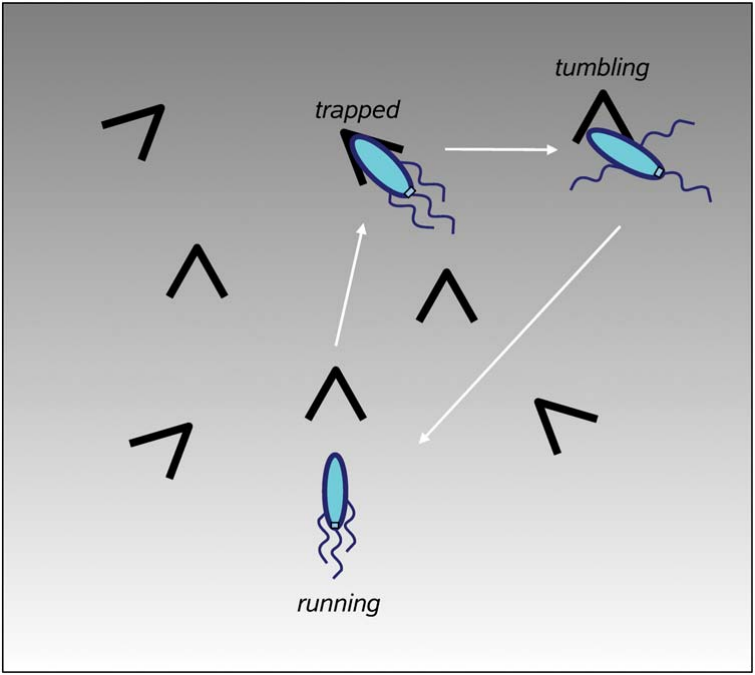
Model of motility in a porous medium (agar). A cell encounters traps along its run, and stops in the traps. It stays in the trapped state until the first tumble occurs, then normal run and tumble behavior resumes. The trap positions are not fixed in the 2D space - instead, it is assumed that each cell encounters traps in a series of randomly distributed time intervals.

In swarm assays, bacteria move in a labyrinth of agar filaments, with obstacles and traps along the cell's path. The cell can encounter traps during its run, and stays trapped until it makes the next tumble, as observed by Wolfe and Berg [55]. Therefore, non-adapting cells and non-tumbling mutants form the smallest rings. To simulate motility in such a porous medium as agar, we have introduced a new state of the cell, corresponding to a stop in a trap during a run (Figure 9). The positions of traps are not fixed in space. Instead, it is assumed that each cell encounters traps in an exponentially distributed time series, which mimics the random collisions of the cell with agar filaments. The mean free time between traps is set to 2.0 s to achieve biologically realistic drift velocities (about 1 *µm* *s* ^−1^). While it is trapped, the cell remains stationary until it makes a tumble, whereupon normal run and tumble behavior resumes until the next stop occures [55].

### Optimal [CheR,CheB] in Agar—Experiments and Simulations

In our model, we assumed that the levels of the adaptation enzymes CheR and CheB vary in a coordinated manner, leaving the [CheR]/[CheB] ratio the same as in the wild type. The ratio of CheR to CheB can be assumed to remain largely fixed because their genes are adjacent and transcriptionally coupled in the *meche* operon. The adaptation rate in our model is thus proportional to the level of co-expression of CheR and CheB, which will be further denoted as [CheR,CheB].

In order to study chemotactic efficiency at different adaptation rates in agar, we have experimentally measured chemotactic efficiency on swarm plates. In these experiments, CheR and CheB-YFP were co-expressed from one operon under control of a pBAD promoter and native ribosome-binding sites. The pBAD promoter gives expression levels lower or higher than the wild-type value, depending on the strength of arabinose induction. Mean protein levels in the population at a given induction were determined as described in Experimental Methods.

**Figure 10:**
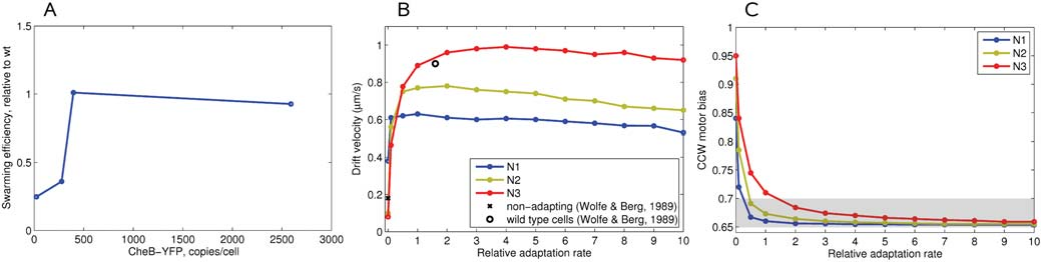
Swarm-plate assay at different [CheR,CheB]. (A) Experimentally measured chemotactic efficiency at different expression levels of the *cheR cheB-eyfp* operon under the control of a pBAD promoter. The applied arabinose concentrations were 0.0, 0.0005, 0.001, 0.01%, respectively. The CheB-YFP level reflects the concerted [CheR,CheB-YFP] due to strong translational coupling. For scale conversion, the wild-type level of CheB can be taken as 240 copies/cell [35]. (B) Simulated chemotactic efficiency as a function of [CheR,CheB]. Cells are simulated in the constant-activity gradients N1 (blue), N2 (green), N3 (red). The black open circle shows the experimentally observed drift velocity of wild-type cells, estimated from Figure 4 of [55]. The cross shows the drift velocity of non-adapting cells, from Figure 6 of [55]. The cell parameters are as described in Table 1. (C) Average motor bias of cells as a function of [CheR,CheB]. The steady-state motor bias is 0.65, with the gray band indicating the region of optimal motor bias for chemotaxis in agar.

Experiment and simulations show that cells with [CheR,CheB] above a certain threshold perform chemotaxis equally efficiently (Figure 10A and 10B). However, the cells with [CheR,CheB] below the threshold have severely impaired chemotactic behavior. According to the simulations, cells with low [CheR,CheB] tend to run without tumbling and stay trapped most of the time. On the other hand, cells with extremely high [CheR,CheB] loose their sensitivity to the gradient and also have poor chemotactic efficiency (Figure S5).

This suggests a positive selection for cells with optimal [CheR,CheB] in liquid media—such cells can reach the nutrient source faster and have more available substrates for growth. In contrast, swimming in agar poses mainly negative selection—cells with low [CheR,CheB] are filtered out from the chemotactic population. The limits of motor bias for optimal chemotaxis in agar are also different from those in liquid media. As one can see in Figure 10C, the average CCW motor bias of successful cells is just slightly higher than the steady-state *mb*_0_. Cells with higher motor bias would drift faster in liquid media, but not in agar, because the period of time they remain trapped also increases with CCW motor bias.

### Swimming in a Liquid Medium and Agar with a Log-Normal Distribution of [CheR,CheB]

To model swarm assays more realistically, we simulated cell populations with a log-normal distribution of [CheR,CheB] values. The mean (1.6) and standard deviation (0.48) are fitted to reproduce the variability of adaptation times observed for wild-type cells [33]: *T_ad_* = 311±150 s in response to a 0–10^−3^ M MeAsp step.

**Figure 11:**
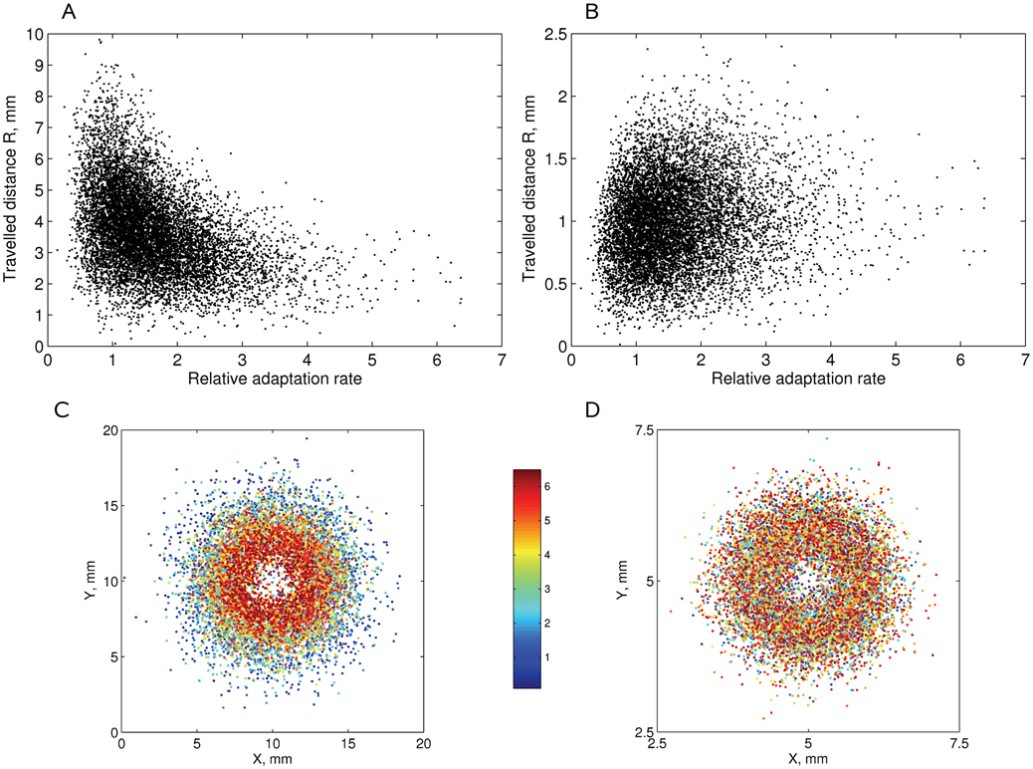
Simulation of motility in a liquid medium and agar with a physiological [CheR,CheB] distribution. The distances *R* travelled by 10^4^ cells after 1000 s of simulation time in (A) the liquid medium, N2 gradient; (B) agar, N3 gradient. The (x,y)-positions of cells colored from deep blue to red, according to their [CheR,CheB], are shown in (C) for the liquid medium, (D) for agar. The smallest [CheR,CheB] values correspond to deep blue, the highest values correspond to red. Note the different scales of the figures. The cell parameters are as described in Table 1.

The scatter plot of distances travelled by cells along the gradient N2 in a liquid medium shows that a subpopulation with optimal [CheR,CheB] levels drifts more rapidly than other cells (Figure 11A). Simulations in the N3 gradient in agar show that cells with low [CheR,CheB] levels are hindered by agar traps, while other cells drift successfully (Figure 11B). In Figure 11C and 11D the same cells are colored from deep blue to red, according to their [CheR,CheB]. The outer edge of the bacterial ring in a liquid medium contains many blue cells with [CheR,CheB] between 0.5 and 2. In contrast, the outer edge in the agar contains a uniform mixture of cells with different [CheR,CheB] levels, while deep blue cells with low [CheR,CheB] tend to be left behind.

### Measurement of [CheR,CheB] in Individual Cells in Different Parts of Swarm Rings

**Figure 12:**
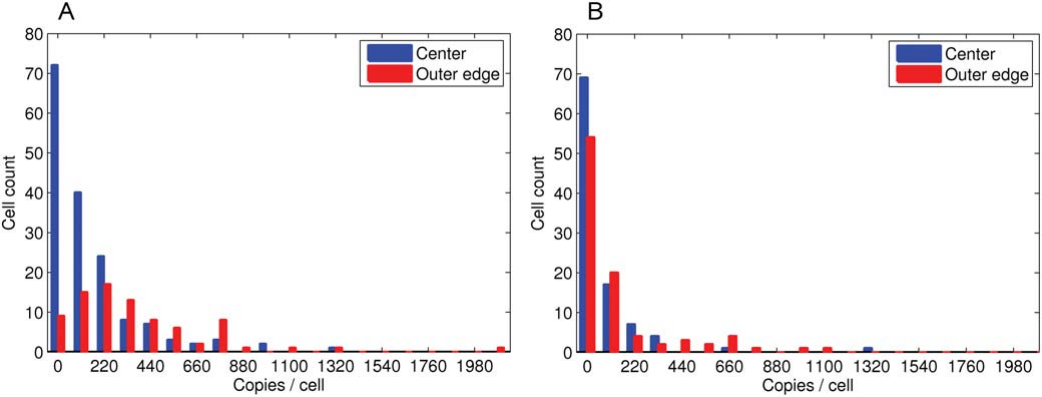
Experimental measurement of [CheR,CheB-YFP] in individual cells at different points in the swarm ring, for plates with (A) normal agar (0.27%); (B) liquid agar (0.20%). Blue columns show the least swarming cells in the center of the swarm plate, and the red ones—the best swarming cells from the outer edge. The expression of *cheR cheB-yfp* was under the control of a pBAD promoter, which gives a basal expression level close to wild-type. The bin size is 110 copies/cell.

To confirm that chemotactic cells are selected for their [CheR,CheB] levels in swarm plates, cells expressing CheR and CheB-YFP from one operon were taken from two positions in the swarm ring—at the center and at the outer edge—and protein levels in individual cells were determined using fluorescence imaging. The cells collected near the center at a standard agar concentration (0.27%) have on average lower copy numbers of adaptation enzymes than cells at the outer edge, confirming the predicted selection against low copy numbers (Figure 12A). As expected, in the swarm plates with a reduced agar concentration (0.20%), the difference between center and outer edge is much smaller (Figure 12B), suggesting that there is no strong selection against low copy numbers in liquid media. It should be noted that agar concentrations below 0.20% do not produce a stable gel structure, and therefore that is probably the most liquid agar that can be used for swarm plate experiments.

Our simulations and additional experiments with a pTrc promoter, which gives much higher basal expression level of [CheR,CheB], show that very high levels of the adaptation enzymes, over 20-fold, can again decrease chemotactic efficiency in agar (Figures S5 and S6).

## Discussion

In this paper, we present RapidCell—a model of chemotactic *E. coli*, which allows us to study the effect of chemotaxis network properties on the behavior of large bacterial populations. RapidCell uses a hybrid model for pathway simulation, with mixed algebraic and ODE description instead of a fully stochastic model, AgentCell [19], or a complete system of ordinary differential equations, *E. solo* [23]. Our model allowed us to dramatically decrease in computational costs. Though many molecular details are skipped or modeled in a rapid-equilibrium (algebraic) approximation, the key steps of the network are reproduced in agreement with up-to-date experimental data. In contrast to detailed single-cell simulation programs which reproduce the noisy behavior of individual cells [19],[56], RapidCell is aimed at predicting the averaged behavior of bacterial populations, and to investigate how it is affected by the signaling network parameters, neglecting the intrinsic noise coming from molecular reactions. However, artificial sources of noise can be further added in the deterministic model of the signaling pathway. In the present version of RapidCell, the noise arises only from rotational diffusion and stochastic switching of the motors.

For the receptor cluster simulation, we used the mixed-receptor cluster MWC model [12],[28],[30], which accounts for the observed broad range of sensitivity and reproduces the recent *in vivo* FRET data [27]. Adaptation is modeled according to the mean-field approximation of the assistance-neighborhood model, with the assumption that the average methylation level of multiple receptors can be represented as a continuous rather than a discrete variable [30]. In contrast to the other reactions, methylation and demethylation are relatively slow and therefore described by an ODE. The ODE is integrated by the first-order Euler scheme to ensure high computational speed of the program, while the time step is chosen as 0.01 s to keep the simulation error low.

Taking into account the available experimental studies on tumble mechanics [31],[57], we use a voting model of run-tumble switching [13],[31],[44]. The model is in a good agreement with experimentally measured run and tumble times. However, more high-resolution experimental data on the interplay among multiple flagella during the run and the tumble would be necessary for a detailed model of run-tumble cellular behavior.

There are several types of gradients usually applied in computer models of chemotaxis. The linear gradient arises between stationary source and adsorber, and can often be observed under natural conditions. The Gaussian, another commonly used gradient, appears when a limited amount of molecules is injected into the medium from a micropipette or a similar source [42]. Other gradients that arise from general models of diffusion have hyperbolic or exponential shapes. However, all commonly used gradients have a ‘blind’ zone where receptors are saturated and cells do not respond. When cells drift along these gradients, the average profile of CheY-P changes dramatically, from a steep fall at low concentrations to a weakly stimulated state at high concentrations (Figure 3C). This makes it difficult to compare long-term chemotactic efficiency, because the average CheY-P and drift velocity are non-stable along the gradient.

To study chemotaxis systematically, we propose a new—constant-activity—type of gradient. This gradient has the unique property of providing the same CheY-P level and cellular-drift velocity over a wide range of ligand concentrations. The stability of the CheY-P level allows us to study properties of virtual chemotactic cells systematically, and to compare chemotactic behavior over long time periods and concentration ranges.

The form of the constant-activity gradient is derived from the MWC model, by formulating the differential equation for the gradient shape which will give a constant rate of receptor free energy change due to ligand binding. In earlier work, the condition of constant chemotactic response was studied using a phenomenological model of ligand binding, with a single dissociation constant *K_D_* [48]. The study of Block and co-authors showed that such a model can be simplified, and as a result an exponential ramp of ligand should give a constant response in the range between *C_min_* = 0.31*K_D_* and *C_max_* = 3.2*K_D_*, a prediction that was supported by their experiments [48].

In our study, we show that the differential equation for the constant-response gradient proposed in [48] is the result of the MWC model. We further solve this differential equation analytically, and find the exact form of the constant-activity gradient. This gradient grows similarly to the exponential function at moderate ligand concentrations, and increases faster than exponential at low and high concentrations (Figure 2A).

Our simulations show that the chemotactic response of the MWC model in the constant-activity gradient remains stable over four orders of ligand concentration—between 0.1 and 1000*K_D_*, in the case when Tsr receptors are fully insensitive to the ligand. However, in the case of (Me)-Asp, the Tsr receptors are able to respond non-specifically to high ligand concentrations, therefore above 100*K_D_* the cluster activity drops to zero in a mixed-receptor cluster [12],[27]. However, our simulations of population behavior consider only moderate Asp concentrations, so the cluster activity remains nearly constant in all observed cases.

The exponential ramp also gives nearly constant response in the MWC model, but over a much smaller range—between 0.5 and 3.0*K_D_*, in agreement with [48] and the recent study of Tu et al. [58].

We also show that the apparent dissociation constant *K_D_* can be estimated by either the arithmetic or geometric mean of *K^off^* and *K^on^*, but the geometric mean gives a better approximation over a wide range of ligand concentrations.

The shape of the constant-activity gradient is also close to a hyperbolic gradient, with the change of variables, *K_D_Cx*/(1−*Cx*) = *K_D_*(1/*y*−1)∼*K_D_*/*y*, (*y* = 1−*Cx*, *K_D_*≪1). The hyperbolic gradient arises from simple models of diffusion, when ligand molecules are emitted from a spherical source into the surrounding medium. In nature, such conditions can be observed, for example, in aquatic ecosystems where microalgae leak organic matter attractive for bacteria [59]. This suggests that hyperbolic and exponential gradients with appropriate parameters can be good approximations for the constant-activity gradient.

In our model, the adaptation rate is assumed to be proportional to the co-varied concentration of the adaptation enzymes [CheR,CheB], and we use both terms to denote the rate of adaptation. However, increasing expression of the adaptation enzymes may lead to saturation of the adaptation rate at some point, because the enzymes will start working out of saturation kinetics. For these reasons, it is more correct to consider our results in terms of adaptation-rate effects on chemotaxis, whatever the origins of adaptation-rate variability may be.

The effect of adaptation rate on chemotaxis agrees in many respects with the results reported in [13] for optimal noise filtering of the chemotaxis signaling system. In their work, the authors demonstrated the existence of an optimal cutoff frequency, an analog of the adaptation rate in our study, for efficient chemotaxis. For a fixed linear gradient, they show the same shape of chemotactic efficiency as a function of cutoff frequency (Figure 3B in [13]) as we found in our simulations (Figure 5A). The authors also show that the optimal cutoff frequency depends on gradient steepness in a linear manner (Figure 5A in [13]), consistent with our results (Figure 5B) for steep gradients.

Our simulations in the constant-activity gradient suggest a simple biological mechanism that determines the optimal adaptation rate for a given gradient steepness. Different optimal adaptation rates correspond to a single CheY-P interval, which fits the linear range of the motor-response function. This means that the highest drift velocity in liquid media is observed when the CheY-P level is in the narrow interval fitting the operating range of the motor. In this range, the dependence between CheY-P and *mb* is approximately linear (Figure 6C).

We found that the CheB phosphorylation feedback can have either a positive or negative effect on chemotactic efficiency, depending on how it shifts the average CheY-P level relative to the region of linear motor response. In the case of non-perfect ratio of CheR to CheB, the CheB phosphorylation mechanism can partially counteract the negative effect of unbalanced [CheR]/[CheB], by shifting the average CheY-P towards the optimal region. This confirms that CheB phosphorylation can improve the chemotactic properties of cells with deviations in the ratio of [CheR]/[CheB], as well as in the ratios of other proteins, from the optimum [32].

Chemotactic behavior in liquid media differs from that in agar. We simulated agar effects using traps randomly distributed over time - a cell can encounter traps during its run, and stays trapped until it makes the next tumble, as observed by Wolfe and Berg [55]. This restricts cellular motility—cells that are highly biased towards running remain in traps longer. In agar, the region of optimal motor bias is very narrow and is just above the unstimulated state *mb*_0_, because higher bias increases the period of time cells remain in traps.

In our model, we did not take into account the growth of a bacterial populations. The typical swarm plate experiments last several hours, and cells grow and divide during the experiment, leading to variations in protein levels and to redistribution of proteins from generation to generation. However, the effect of different adaptation rates in our simulations is clearly visible already within one cell generation over 1000 s of model time (Figure 11B). The selection thus works on a time scale that is shorter than the generation time, which, in our opinion, justifies using a fixed protein distribution. Therefore, the addition of cell growth should not change our results qualitatively. In experiments, daughter cells with sub-optimal levels of CheR and CheB will rapidly fall behind the spreading swarm ring in the vicinity of the division site, while the subpopulation with optimal adaptation rates will be always at the front edge of the ring.

In most of our simulations, we assume that the CheR and CheB ratio is constant due to the genetic coupling between the two respective genes, and that cell-to-cell variation in adaptation rates arises from concerted variation in the levels of both enzymes [32]. We also investigated the effects of variation in the [CheR]/[CheB] ratio, which results from translational noise, and affect both the adaptation rate and the steady-state motor bias. In addition to these investigated sources of noise, there is intrinsic noise in the pathway activity which arises from the stochastic nature of (de-)methylation events. The latter sort of noise can also have positive effects on the spreading of cells in a ligand-free medium [56], and even on chemotactic drift in weak gradients [60]. Superposition of variable noise effects on chemotactic efficiency in variable gradients would be an interesting issue for further study.

In this work, we have estimated the variability in concerted CheR and CheB concentrations using available experimental data on cell-to-cell variability in adaptation times [33]. We assumed a log-normal distribution for protein concentrations, which also gives a log-normal distribution of adaptation times to a step-wise stimulus from 0 to 10^−3^ *M* MeAsp [33]. There are also other experimental estimates of cell-to-cell variation in adaptation times [34] and related simulations [61], but the adaptation rates observed in those experiments were several times higher, presumably due to different culture growth conditions.

Our simulations suggest some evolutionary implications. In liquid media with variable food sources and gradient intensities, variability in adaptation times (protein levels) among cells can help the whole population to respond to different gradients more readily, due to positive selection of cells with optimal [CheR,CheB]. In other words, for any given gradient steepness, there will be a subpopulation which has the best [CheR,CheB] to follow this gradient. In contrast, agar poses mainly negative selection on cell populations - cells with low [CheR,CheB] are filtered out from competition, while all other cells travel with approximately equal efficiency.

Inspired by the implementation of AgentCell, RapidCell focuses on highly efficient computation of large populations over long periods, keeping cell-response properties consistent with experimental data. The first version of RapidCell allows us to simulate *E. coli* populations of size 10^4^–10^5^ cells over a time scale of hours, while tracking the signal network dynamics of individual cells. These capabilities permit the modeling of cellular behavior on a macroscopic scale, as in swarm-plate experiments, and the prediction of properties of heterogeneous populations with noisy components of the signaling network.

## Supporting Information

Figure S1 Comparison of the RapidCell network response with experimental and simulated data. (A) FRET experiment and RapidCell simulation of cell response to a step-wise stimulus of MeAsp. The initial ambient concentration is zero; at t = 80 s 30 µM MeAsp is added and removed at 480 s. The best fit by RapidCell is obtained with an adaptation rate of k = 0.5, corresponding to the temperature T = 20°C at which the FRET experiments were carried out. At T = 30°C, the fitted adaptation rate will be k = 1.0 (V.Sourjik, unpublished data). (B) StochSim and RapidCell simulations of cell response to a step-wise stimulus of Asp. The initial ambient concentration is zero; at t = 20 s 3.5 µM Asp is added and removed at 70 s. The best fit by RapidCell is obtained with an adaptation rate of k = 8 - a very rapid rate of adaptation. The StochSim simulations were carried out with a coupled model (Shimizu et. al, 2003), consisting of 65×65 square receptor lattice with coupling energy E_J_ = −3.1 kT.
(0.30 MB TIF)

Figure S2 Comparison of the RapidCell network response with experimental data on tethered cells. (A) Simulation of CCW motor bias response to a short pulse of attractant. The initial ambient concentration is zero; at t = 5 s 1.0 mM Asp is added for a 0.35 s interval; solid line - simulations (the best fit is obtained with an adaptation rate of 2.0), circles - experimental data (Segall et. al., 1986). (B) Simulation of CCW motor bias response to a step-wise stimulus. The initial ambient concentration is zero; at t = 1 s 0.075 µM Asp is added; solid line - simulations, circles - experimental data (Segall et. al., 1986). The best fit is obtained with an adaptation rate of 5.0. (C) Adaptation times to a step increase of MeAsp from zero ambient level, obtained in simulations (solid line) and in experiments (Berg and Tedesco, 1975) (circles). In the simulations, the dissociation constants used were K_a_^off^ = 0.02 mM and K_a_^on^ = 0.5 mM (Keymer et. al., 2006). The best fit is obtained with an adaptation rate of 1.3.
(0.06 MB TIF)

Figure S3 Probability density function of tumbling angles f(Θ) = 0.5(1+CosΘ)SinΘ used in the model (solid line), and experimental measurements (cross markers) (Berg and Brown, 1972).
(0.04 MB TIF)

Figure S4 The CheY-P response of the MWC model to the constant-activity ramp of aspartate from 0.1 to 10000K_D_. The ramp is simulated according to Eqn. 22 in two forms, with K* = 0.5(K^on^+K^off^) (arithmetic mean), or K* = (K^on^K^off^)^0.5^(geometric mean). The MWC model shows an approximately constant response for both approximations, but the geometric mean gives the more stable response over a wider range of concentrations.
(0.12 MB TIF)

Figure S5 Chemotactic efficiency in agar as a function of highly over-expressed [CheR,CheB], observed in experiments and simulations: (black line) swarm-plate efficiency of cells with CheR and CheB-YFP expression under the control of a pTrc promoter. The chemotactic efficiency was estimated relative to the diameters of wild-type swarm rings. Color lines denote simulated chemotactic efficiency in three constant-activity gradients N1 (blue), N2 (green), N3 (red). The chemotactic efficiency in the simulations was estimated as the average distance travelled by cells, divided by the distance with the optimal [CheR,CheB]. Error bars indicate standard deviations.
(0.06 MB TIF)

Figure S6 Measurement of [CheR,CheB] in individual cells in different points of the swarm ring, for cells with (A) the least, and (B) the best swarming efficiency. CheR and CheB-YFP were expressed from one operon under the control of a pTrc promoter and native ribosome-binding sites. The pTrc promoter gives high basal expression relative to the wild-type level. The least swarming cells were taken from the center of the swarm plate, and the best swarming - from the outer edge of the swarm ring. The mean protein levels were determined as described in Experimental Methods.
(0.06 MB TIF)

Table S1 Rates of reactions
(0.02 MB PDF)
