# Supplementary figures and images for: Dependence of Bacterial Chemotaxis on Gradient Shape and Adaptation Rate
Source: PLoS Comput Biol. 2008 Dec 19;4(12):e1000242. doi: 10.1371/journal.pcbi.1000242 (PMC2588534; doi:10.1371/journal.pcbi.1000242)

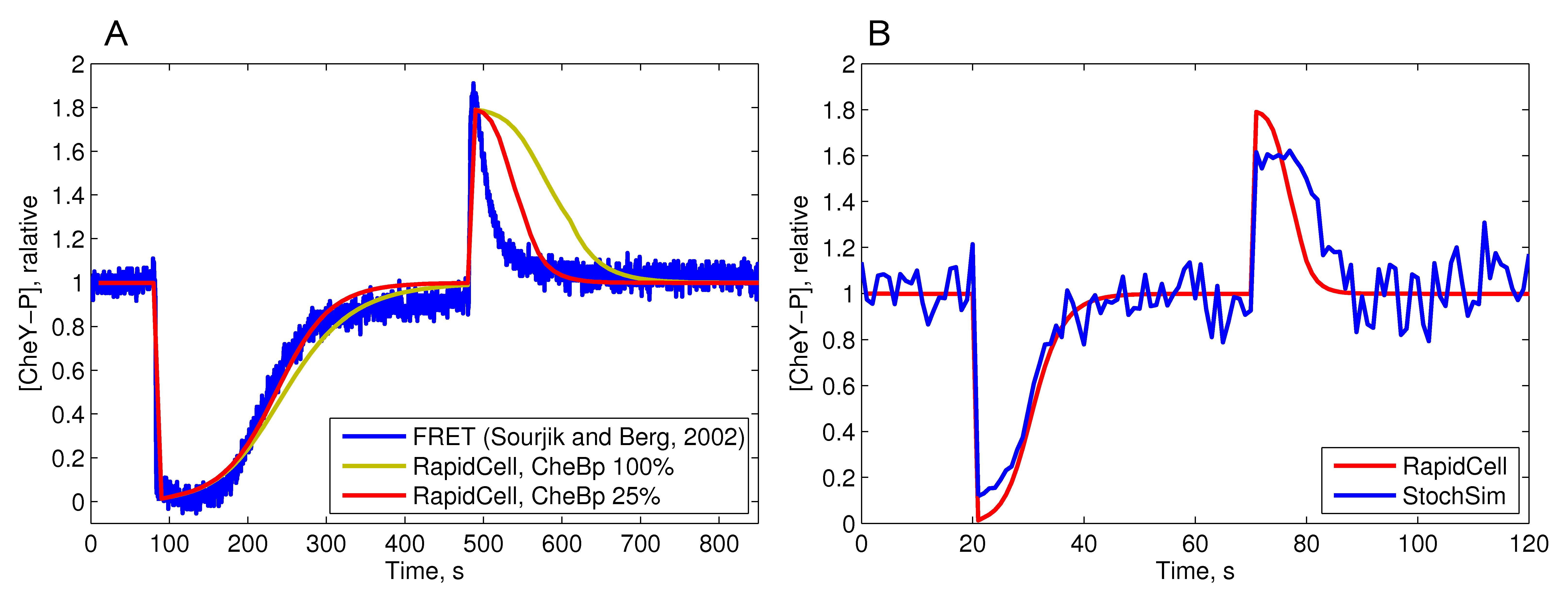

Supplement: Figure S1 — Comparison of the RapidCell network response with experimental and simulated data. (A) FRET experiment and RapidCell simulation of cell response to a step-wise stimulus of MeAsp. The initial ambient concentration is zero; at t = 80 s 30 µM MeAsp is added and removed at 480 s. The best fit by RapidCell is obtained with an adaptation rate of k = 0.5, corresponding to the temperature T = 20°C at which the FRET experiments were carried out. At T = 30°C, the fitted adaptation rate will be k = 1.0 (V.Sourjik, unpublished data). (B) StochSim and RapidCell simulations of cell response to a step-wise stimulus of Asp. The initial ambient concentration is zero; at t = 20 s 3.5 µM Asp is added and removed at 70 s. The best fit by RapidCell is obtained with an adaptation rate of k = 8 - a very rapid rate of adaptation. The StochSim simulations were carried out with a coupled model (Shimizu et. al, 2003), consisting of 65×65 square receptor lattice with coupling energy EJ = −3.1 kT. (0.30 MB TIF) [file pcbi.1000242.s001.tif]

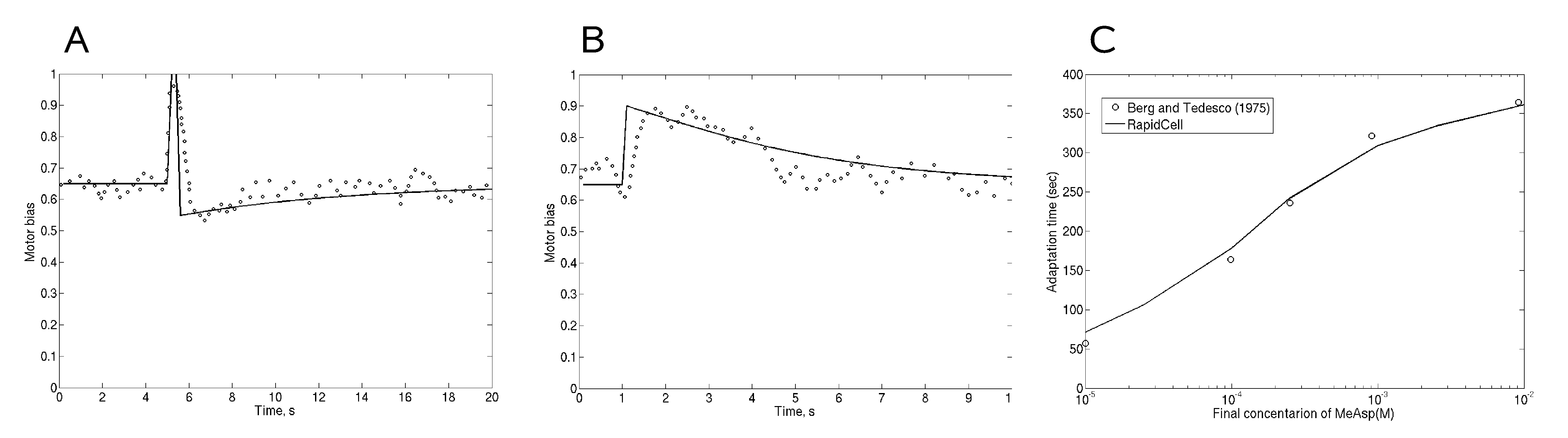

Supplement: Figure S2 — Comparison of the RapidCell network response with experimental data on tethered cells. (A) Simulation of CCW motor bias response to a short pulse of attractant. The initial ambient concentration is zero; at t = 5 s 1.0 mM Asp is added for a 0.35 s interval; solid line - simulations (the best fit is obtained with an adaptation rate of 2.0), circles - experimental data (Segall et. al., 1986). (B) Simulation of CCW motor bias response to a step-wise stimulus. The initial ambient concentration is zero; at t = 1 s 0.075 µM Asp is added; solid line - simulations, circles - experimental data (Segall et. al., 1986). The best fit is obtained with an adaptation rate of 5.0. (C) Adaptation times to a step increase of MeAsp from zero ambient level, obtained in simulations (solid line) and in experiments (Berg and Tedesco, 1975) (circles). In the simulations, the dissociation constants used were Ka off = 0.02 mM and Ka on = 0.5 mM (Keymer et. al., 2006). The best fit is obtained with an adaptation rate of 1.3. (0.06 MB TIF) [file pcbi.1000242.s002.tif]

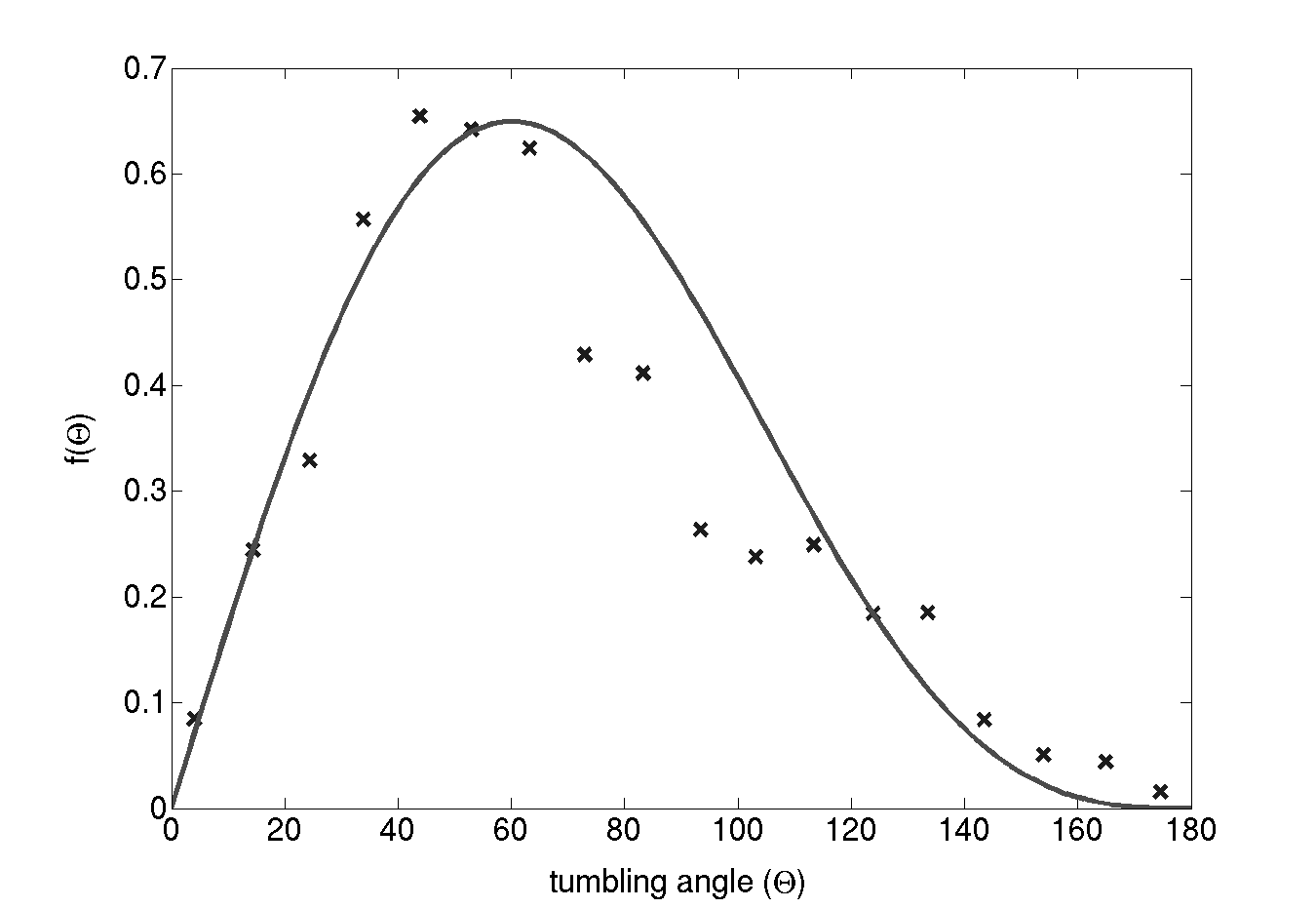

Supplement: Figure S3 — Probability density function of tumbling angles f(Θ) = 0.5(1+CosΘ)SinΘ used in the model (solid line), and experimental measurements (cross markers) (Berg and Brown, 1972). (0.04 MB TIF) [file pcbi.1000242.s003.tif]

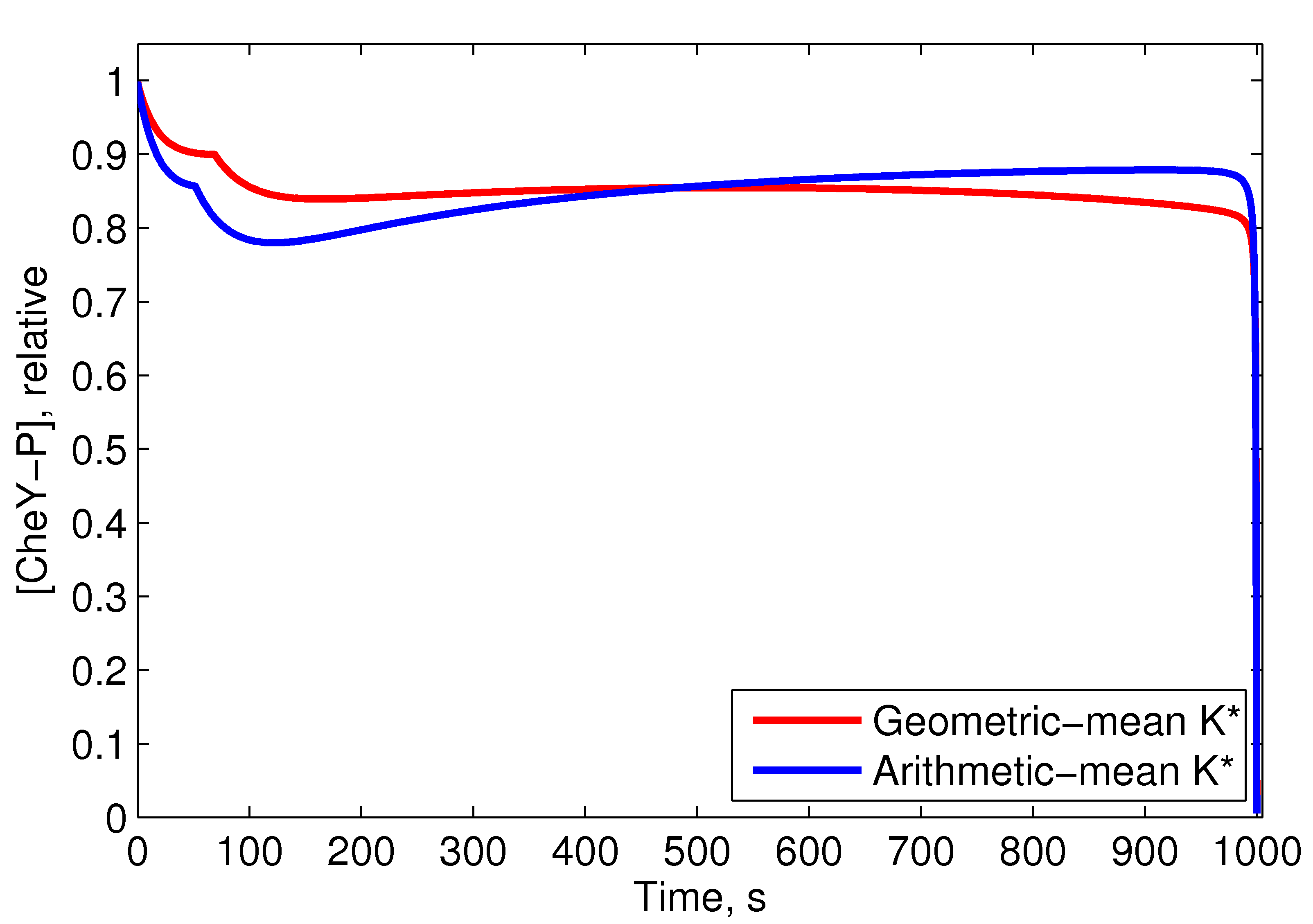

Supplement: Figure S4 — The CheY-P response of the MWC model to the constant-activity ramp of aspartate from 0.1 to 10000KD. The ramp is simulated according to Eqn. 22 in two forms, with K* = 0.5(Kon+Koff) (arithmetic mean), or K* = (KonKoff)0.5(geometric mean). The MWC model shows an approximately constant response for both approximations, but the geometric mean gives the more stable response over a wider range of concentrations. (0.12 MB TIF) [file pcbi.1000242.s004.tif]

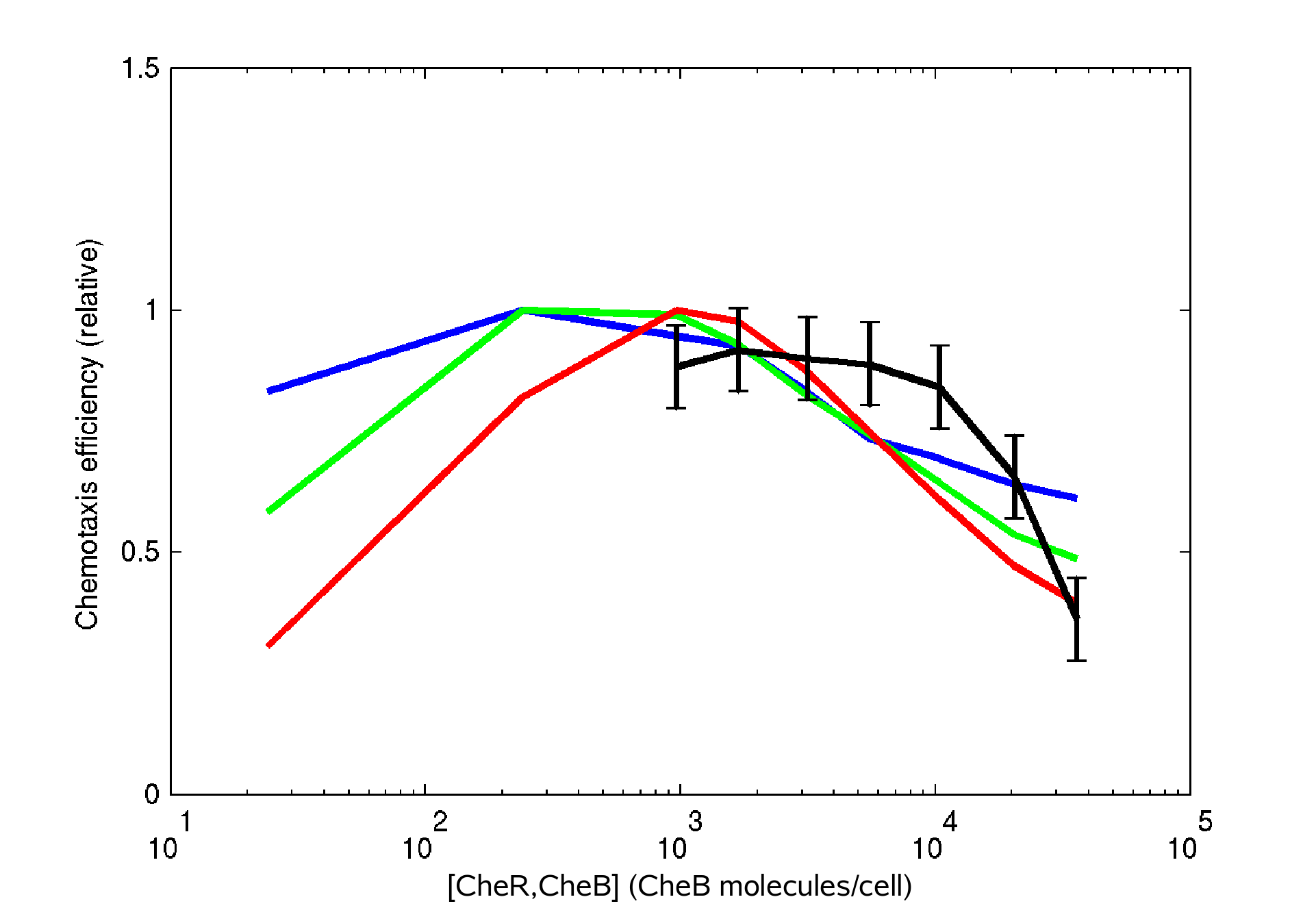

Supplement: Figure S5 — Chemotactic efficiency in agar as a function of highly over-expressed [CheR,CheB], observed in experiments and simulations: (black line) swarm-plate efficiency of cells with CheR and CheB-YFP expression under the control of a pTrc promoter. The chemotactic efficiency was estimated relative to the diameters of wild-type swarm rings. Color lines denote simulated chemotactic efficiency in three constant-activity gradients N1 (blue), N2 (green), N3 (red). The chemotactic efficiency in the simulations was estimated as the average distance travelled by cells, divided by the distance with the optimal [CheR,CheB]. Error bars indicate standard deviations. (0.06 MB TIF) [file pcbi.1000242.s005.tif]

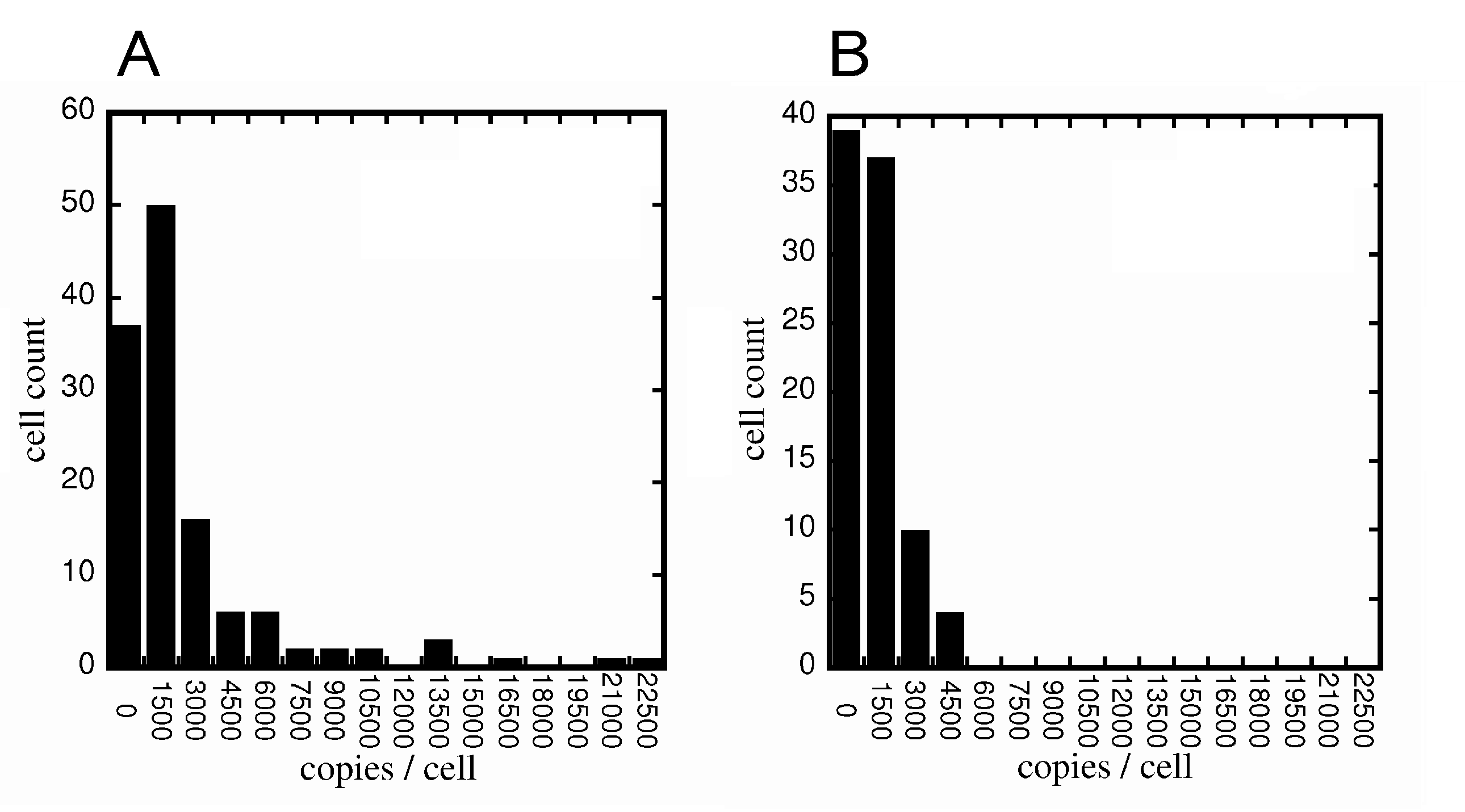

Supplement: Figure S6 — Measurement of [CheR,CheB] in individual cells in different points of the swarm ring, for cells with (A) the least, and (B) the best swarming efficiency. CheR and CheB-YFP were expressed from one operon under the control of a pTrc promoter and native ribosome-binding sites. The pTrc promoter gives high basal expression relative to the wild-type level. The least swarming cells were taken from the center of the swarm plate, and the best swarming - from the outer edge of the swarm ring. The mean protein levels were determined as described in Experimental Methods. (0.06 MB TIF) [file pcbi.1000242.s006.tif]
