# Supplementary material for: Dependence of Bacterial Chemotaxis on Gradient Shape and Adaptation Rate
Source: PLoS Comput Biol. 2008 Dec 19;4(12):e1000242. doi: 10.1371/journal.pcbi.1000242 (PMC2588534; doi:10.1371/journal.pcbi.1000242)
Supplement: Table S1 — Rates of reactions (0.02 MB PDF) [file pcbi.1000242.s007.pdf]

Description	Value [1/s]		
	ref. (1)	ref. (2)	ref. (3)
CheR catalytic rate	0.819	0.39	0.75
CheB catalytic rate	0.155	6.3	0.6
CheA autophosphorylation rate	15.5	50	23.5
CheY phosphorylation rate	15	530	530
CheB phosphorylation rate	15	15.9	53
CheY dephosphorylation rate	14.5	30.1	30

Supplementary Table S1.

Rates of reactions involved in the signaling pathway, according to (1) Morton-Firth et al, 1999; (2) Kollmann et al., 2005; (3) Emonet and Cluzel, 2008.
